# German translation and pre-testing of Consolidated Framework for Implementation Research (CFIR) and Expert Recommendations for Implementing Change (ERIC)

**DOI:** 10.1186/s43058-021-00222-w

**Published:** 2021-10-19

**Authors:** Verena Regauer, Eva Seckler, Craig Campbell, Amanda Phillips, Thomas Rotter, Petra Bauer, Martin Müller

**Affiliations:** 1 Centre for Research, Development and Technology Transfer, Rosenheim Technical University of Applied Sciences, Hochschulstraße 1, 83024 Rosenheim, Germany; 2 Institute for Medical Information Processing, Biometry and Epidemiology, Ludwig-Maximilians-Universität München, Marchioninistraße 17, 81377 Munich, Germany; 3 University of Saskatchewan Language Centre, 221 Cumberland Avenue North, Saskatoon, Saskatchewan S7N 1M3 Canada; 4 School of Nursing, Queen’s University, 82-84 Barrie Street, Kingston, Ontario K7L 3N6 Canada; 5 Faculty for Applied Health and Social Sciences and Centre for Research, Development and Technology Transfer, Rosenheim Technical University of Applied Sciences, Hochschulstraße 1, 83024 Rosenheim, Germany

**Keywords:** Implementation science, Implementation strategies, Knowledge translation strategies, Consolidated Framework for Implementation Research, Expert Recommendations for Implementing Change

## Abstract

**Background:**

Implementation frameworks may support local implementation strategies with a sound theoretical foundation. The *Consolidated Framework for Implementation Research (CFIR)* facilitates identification of contextual barriers and facilitators, and the *Expert Recommendations for Implementing Change (ERIC)* allows identifying adequate implementation strategies. Both instruments are already used in German-speaking countries; however, no standardised and validated translation is available thus far. The aim of this study was to translate the CFIR and ERIC framework into German, in order to increase the use of these frameworks and the adherence to evidence-based implementation efforts in German-speaking countries.

**Methods:**

The translation of the original versions of the CFIR and ERIC framework was guided by the World Health Organisation’s recommendations for the process of translating and adapting both conceptual frameworks. Accordingly, a four-step process was employed: first, forward translation from English into German was conducted by a research team of German native speakers with fluent knowledge of the English language. Second, a bilingual expert panel comprising one researcher with German as his mother tongue and expert command of the English language and one English language expert and university teacher reviewed the translation and discussed inconsistencies with the initial translators. Third, back-translation into English was conducted by an English native speaking researcher. The final version was pre-tested with 12 German researchers and clinicians who were involved in implementation projects using cognitive interviews.

**Results:**

The translation and review process revealed some inconsistencies between the original version and the German translations. All issues could be solved by discussion. Central aspects of the items were confirmed in 60 to 70% of the items, and modifications were proposed in 30% of the items. Finally, we revised one CFIR-item heading after pre-testing. The final version was given consent by all involved parties.

**Conclusions:**

Now, two validated and tested implementation frameworks to guide implementation efforts are available in the German language and can be used to increase the application of agreed-on implementation strategies into practice.

**Supplementary Information:**

The online version contains supplementary material available at 10.1186/s43058-021-00222-w.

Contributions to the literature

- To increase the application of CFIR and ERIC implementation frameworks in German-speaking countries, these concepts are now available in German
- The conceptual frameworks can be used by clinicians, researchers, managers and organisations to increase adherence to evidence-based implementation activities, and to improve the transferability of this experience into local practice
- The frameworks were translated into German using a standardised WHO approach, including pre-testing with German health care professionals, researchers, and clinicians

## Background

Many interventions in health care are considered to be effective, but efficacy in terms of achieving desired changes in patient-relevant health outcomes is critically dependent on successful implementation [1, 2]. Lack of information about a study’s local context and poor reporting of implementation strategies employed may be accountable for the critical gap between implementation research and clinical practice [2, 3]. The use of implementation frameworks may help to increase adherence to evidence-based implementation strategies and to establish a consensus terminology for German-speaking implementation experts [4].

The *Consolidated Framework for Implementation Research (CFIR)* [1] provides a tool box of different constructs arranged across five domains that should be used in a range of settings. It can help to identify potential barriers and help facilitators to change, and can be used as theory-based constructs for developing effective implementation strategies [1].The *Expert Recommendations for Implementing Change (ERIC)* systematically catalogued implementation strategies via input from a wide range of stakeholders and structured them into different categories and definitions [5, 6].

Identifying barriers and facilitators at different dimensions and tailoring interventions appear crucial to successful implementation of interventions into practice. Moreover, a Cochrane review [7] concluded that tailored interventions addressing implementation barriers are more likely to improve professional practice than untailored interventions, e.g., clinical practice guidelines alone, while more research is needed on the causal mechanisms for successful implementation and how to address these determinants. As a first step for implementation researchers, the CFIR provides a systematic framework to categorise potential barriers and facilitators. As a second step and to tailor the implementation, ERIC catalogues potential implementation strategies. To connect those, a tool was built that linked the context assessment using CFIR and implementation strategies to be considered using ERIC, the *CFIR–ERIC–matching tool* (available under www.cfirguide.org) [8].

Both taxonomies are already used in various studies with a wide range of objectives, methods, and settings [9] including German-speaking countries [10]. However, no standardised and validated translation into German was available thus far.

The aim of this study was to develop a German translation of both CFIR and ERIC in order to increase adherence to evidence-based implementation activities in German-speaking countries.

## Methods

### Study design

**Fig. 1:**
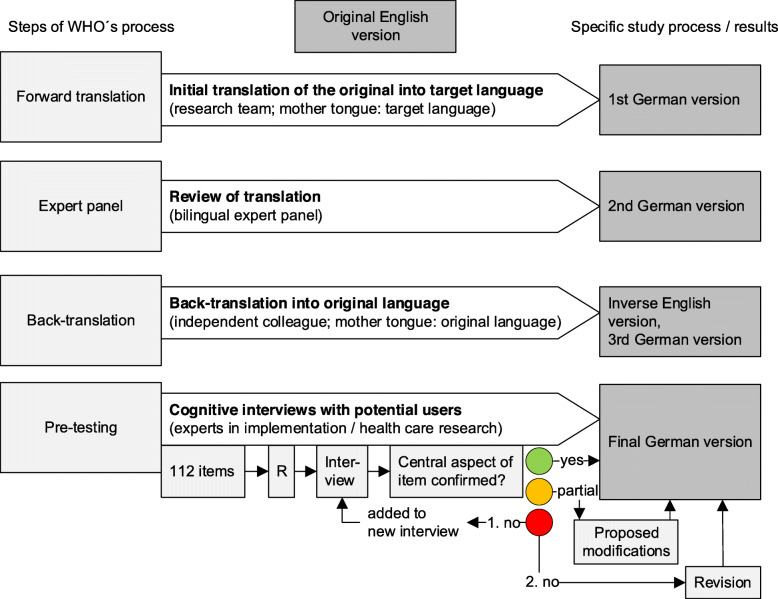
Flowchart describing the WHO translation and pre-testing process [11]. Legend: R = Randomisation

We followed the translation process suggested by the *World Health Organisation (WHO)* [11] that comprises forward translation, expert panel discussion, back-translation, and pre-testing (see Fig. 1).

### Forward translation

The research team, all familiar with the content of CFIR and ERIC and German native speakers with fluent knowledge of the English language, translated the original English version of CFIR [1] and ERIC [6] into German. The aim was to find conceptually equivalent wordings and phrases.

### Expert panel and back-translation

First, a small (unilingual) expert panel with a German-speaking researcher, who already had translated and used a German version of CFIR, and members of the research team was established. Differences between the result of the forward translation of CFIR and the older version were discussed and led to the revision of some items.

Second, a bilingual expert panel discussed both the translation of CFIR and ERIC. The expert panel, which consisted of a collaborating Canadian researcher (TR) with German as his mother tongue and expert command of the English language and one English language expert and university teacher (CC), revised the German translation. They recommended changes of distinct phrases. Inconsistencies were discussed among the research team and the bilingual experts.

The work of the expert panels led to a first German version of CFIR and ERIC.

### Back-translation

Back-translation of the instruments into English was conducted by an independent English native-speaking researcher (AP) living and working in Germany for several years. As recommended by the WHO method, the back-translator had no specific knowledge of the instruments. Inconsistencies between the back-translation and the original versions were discussed among the research team using dictionaries and several online translators. This resulted in the second version of the German translation that went into pre-testing with potential users of the tools.

### Pre-testing

#### Research team

VR and ES conducted the individual, semi-structured interviews with experts working in implementation and/or health care research and who could potentially use the translated instruments. The interviewers knew some of the participants personally. No bias or assumptions are to be reported from the interviewers.

#### Recruitment and design

We conducted individual semi-structured interviews. Potential participants, in particular health care researchers were recruited from universities and research institutes in German-speaking countries and were approached via e-mail. To limit the burden for the participants, we decided to present only a subset of the items to each participant. Considering a total number of 112 items and an estimated interview time of about 2 min for each item, we predefined a sample size of 12 participants to discuss 10 items per 20-min interview. Items of each instrument were randomised to the number of participants using a computer-generated sequence number (Random Sequence Generator, available at https://www.random.org/sequences/). All participants gave written informed consent and filled in a short sociodemographic questionnaire prior to the interview.

#### Data collection and analysis

Since there was no established strategy for pre-testing or validating of these instruments, we had to develop our own strategy. This contains cognitive interviews that involve a “think-aloud-probing” procedure, in which interviewers instruct participants to verbalise thoughts while answering the posed questions [12]. In parts, we relied on the key stages of cognitive interviewing according to Willis et al. [13]. This full strategy comprised five steps.

- Step 1: To warm up with the item during the interview, the interviewees were asked to describe the given definition in their own words.
- Step 2: Interviewees were asked to formulate a heading that describes the content best after reading the detailed definitions. The aim of this step was to generate information about the perceived central focus of those items.
- Step 3: Then, the translated heading in German was compared with the interviewee-suggested heading and discussed afterwards.
- Step 4: To rate this comparison, a traffic light system was used to rate whether our translated heading was perceived as appropriate. In this rating system, “green” means “approved”, “yellow” “partially approved”, and “red” “rejected”. Additionally, text notes were made about why the participants rate “yellow” or “red”.
- Step 5: Items rated “green” were immediately considered to be accepted. In the case of “yellow”, the proposed modifications were recorded and discussed within the research team and adapted if the considered modifications were rated to be meaningful. In the case of ”red”, the item was re-tested in a second interview. When the item was then rated as “green”, it was considered approved. In any other case, we revised the item as recommended by the two interviews and amended the heading with our initial translation in brackets.

Interviews were conducted by phone. Researchers had a short interview guide, and questions were allocated by the randomised items per interviewee. The interview guide was pilot tested with two persons and adapted prior to the interviews. Our initial pilot-tests revealed that rating CFIR barriers together with ERIC strategies was too complex and confusing due to the different foci. Thus, we decided to present items of only one respective framework per interview. In our interviews, we provided the example of implementing an electronic assessment system in a general practice or physiotherapy practice to support participants contextualising implementation items.

No repeated interviews were carried out. All interviews were guided and audio-recorded by one of the two experienced research associate (VR or ES). Field notes were made during the interviews. Audio-records were neither transcribed nor coded. Interviews were recorded to be available as backup for the field notes. Field notes were used to categorise items, and percentages of ratings per interview were calculated. Total percentages were calculated using mean values of percentages for each tool.

### CFIR-ERIC-Matching Tool

We translated the short instructions of the tool into German and contacted the authors. We inserted the final versions of CFIR and ERIC into the matching tool and checked its function.

## Results

### Process

Our initial forward translation of each instrument (see Additional file 1) was revised by the expert panel. Recommendations for changes and our decisions for or against changes are described in Additional file 2. The recommendations included correctness and style like using linguistic synonyms, keeping the ideas of “and/or”, using correct grammar and punctuation marks. For example: CFIR; Item no. 2.3: *Peer pressure*. Initial translation: *Druck durch Kollegen*. Recommendation by the expert panel: *Change “Druck durch Kollegen” into “Gruppenzwang” because this word is more usual*. All issues could be solved by discussion. Then, these first versions were translated back into the original language (see Additional file 3). Then we compared the original English version to the back-translated version, and synonyms were identified. For example: CFIR; Item no. 1.5: *Trialability*. Backtranslation into original language: *Testability*. ERIC; Item no. 61. Original: *Stage implementation scale up*; Backtranslation into original language: *Proceed step by step with the implementation*.

### Cognitive interviews

#### Characteristics of participants

We contacted 12 individuals, all of whom agreed to participate. Mean age of the participants was 36 years, and most of the participants were female (*n* = 10; 83%). All participants had at least a master’s degree, and most were working as research associates (*n* = 10; 83%) and had experience in implementation of health care interventions (see Table 1). The interviews lasted between 9 and 36 min.

**Table 1:** Characteristics of interview participants

	***n*** = 12
Age (years), *mean ± SD*	36.3 ± 7.5
Sex, *female/%*	10/83
Educational level, *n/%*	
Master’s degree	7/58
Doctoral degree	5/42
Professional position, *n/%*	
Research associate	10/83
Other, namely:	
Substitute professorship	1/8
Head of nursing development	1/8
Years of experience in implementation research, *n/%*	
< 2 years	4/33
≥ 2–4 years	3/25
≥ 5–9 years	3/25
≥ 10 years	2/17
Research experience regarding MRC-framework phase (multiple answers possible), *n/%*	
Development	9/75
Feasibility/piloting	8/67
Evaluation	7/58
Implementation	3/25
None of them	1/8
Number of published papers in implementation research, *n / %*	
< 2 papers	8/67
> 2–4 papers	3/25
> 5–9 papers	1/8
> 10 papers	0/0
Working experience using implementation research literature in (multiple answers possible), *n/%*	
Research	10/83
Teaching	6/50
Other, namely: Development of care practice	1/8 1/8
Not at all	1/8

Legend: *SD* standard deviation

#### Findings

In brief, the similar central focus of our German translations of CFIR and ERIC compared to the English original was confirmed in most items.

Among the CFIR items (see Table 2), two items (5%) were rejected in the first round and presented in a second interview. Of these, one item was again rejected, and one was partially approved. In total, 27 items (69%) were approved in the first round. Modifications were proposed for 11 (28%) items. Only one item (3%) had to be revised after pre-testing. Recommendations for modifications can be seen in Additional file 4.

**Table 2:**
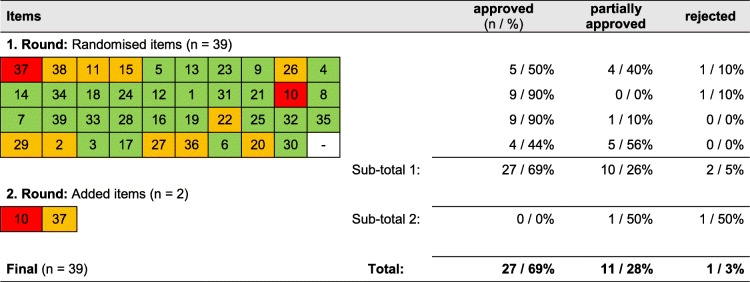
Results of pre-testing CFIR

Legend: *Green* = approved, Yellow = partially approved, *Red* = rejected

Among the ERIC items (see Table 3), two items were rejected in the first round and accepted in the second round. In summary, 47 items (64%) were approved. No item had to be revised. Modifications were proposed for 26 items (36%). Recommendations for modifications can be seen in Additional file 4.

**Table 3:**
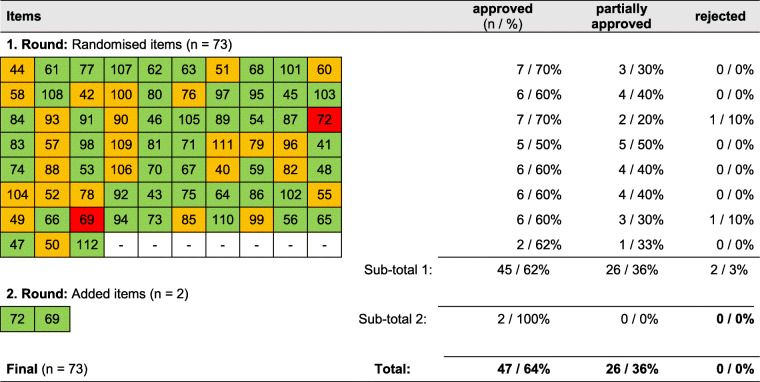
Results of pre-testing ERIC

Legend: *Green colour* = approved, *Yellow* = partially approved; *Red* = rejected

#### Final version

After pre-testing, we revised the rejected item, but kept our pre-tested translation in brackets (see Additional file 5). The final versions were agreed upon by all parties involved in the translation process. A German version of CFIR-ERIC-Matching tool is available upon request.

## Discussion

### Main findings

A German version of two conceptual frameworks for shaping implementation activities in health care, CFIR and ERIC, as well as the corresponding matching tool, are now available.

A rigorous translation and pre-testing process guided us through the WHO translation process to a final version of each framework. The pre-testing process proved to be feasible.

In our initial forward translation, we aimed to keep the original linguistic structure as far as possible. In the expert panel, we discussed alternative translations to items which appeared to be in “Denglish” jargon (a variety of German containing a high proportion of English words), but accepted English words which are common in German (e.g., CFIR, headings 2 and 3, “Äußeres/Inneres Setting”; ERIC, item 35, “Champions identifizieren und vorbereiten”). Beside this, we also tried to keep a coherent structure of the Original and our German translation as possible. Since we referred to Powell et al. [6] in carrying out our forward translation of ERIC, we did not additionally translate the nine categories outlined in Waltz et al. [5]. When using the CFIR-ERIC-matching tool, the categories above the implementation strategies appear not crucial to know, but we would recommend its translation when using ERIC itself in a conceptual context.

Faced with the high number of items, the bilingual expert panels were quite time-consuming. Both ERIC and CFIR documents were very detailed, including health care-specific vocabulary, and nuances that required careful translation, all of which led to taking a lot of time. We recommended a considerable number of changes to the early translation.

Regarding back-translation of the instruments, we predominantly found synonyms to the English original. Close attention was given to the health care-specific vocabulary. This remained a difficult task for the back-translator:

We were not able to identify and use an established strategy for pre-testing or validating these frameworks. A developed checklist in form of a traffic light system to test the usefulness of the translated heading was successful.

The translation and pre-testing process also revealed that even headings in the original version did not comprise the whole content of the detailed definition. For example, CFIR 4.1; Original heading: *Knowledge and Beliefs about the Intervention*; Original description: *Stakeholders have negative attitudes toward the innovation, they place low value on implementing the innovation, and/or they are not familiar with facts, truths, and principles about the innovation*. Pre-tested description: *Beteiligte haben negative Einstellungen gegenüber der Innovation, sie schreiben ihr geringen Wert zu und/oder sind mit den Fakten, Wahrheiten und Prinzipien der Innovation nicht vertraut*; Pre-tested heading: *Wissen und Überzeugungen über die Innovation*; Selected heading by the interviewee: Ablehnung der Innovation; Recommendation of the interviewee: *The negative perception is missing in the original heading*.

## Limitations

During the translation, expert panel and back-translation process, several issues were discussed and kept in the original English language to provide a clear and correct message.

In total, 4 of 112 items were discussed in a second interview. This might indicate selection bias in terms of our interview participants (e.g., allocating an item to “yellow” (partly approved) rather than to “red” (rejected) because participants wanted to be perceived as nice). Since we predominantly tested one item per interviewee, a different sample of participants might have rejected more items. Our sample comprised different researchers and academics experienced in specific terms like “evidence” or “validity”. A pre-test with a different sample of non-academic individuals might have led to different results.

Transferring theoretical frameworks from one language to another is challenging beyond the linguistic perspective but also from the perspective of validity since the contained constructs and relationships between them largely depend on context, such as different health care systems. However, since both frameworks are already being used in German health care, we believe that a standardised and agreed on translated version may increase understanding, uptake, transparency, and reproducibility of implementation research in German speaking countries. Moreover, the standard of English may differ widely between healthcare professionals in Germany.

Future applications of the translated German version of CFIR and ERIC should monitor and report problems and limitations and may lead to further revisions.

## Conclusions

Both translated frameworks can now be used within implementation research in German-speaking countries. This might improve adherence to evidence-based implementation into practice in German-speaking countries. We recommend a patient version of the translated implementation frameworks, which use a lay language (not exceeding German 6th class middle school writing).

## Supplementary Information

**Additional file 1.** Initial forward version

**Additional file 2.** Summary of recommendations by the expert panel

**Additional file 3.** Back-translation

**Additional file 4.** Summary of problems found during the pre-testing of the instrument and the modifications proposed

**Additional file 5.** Final version

## Abbreviations

CFIR: Consolidated Framework for Implementation Research
ERIC: Expert Recommendations for Implementation Change
WHO: World Health Organisation
