# Supplementary material for: German translation and pre-testing of Consolidated Framework for Implementation Research (CFIR) and Expert Recommendations for Implementing Change (ERIC)
Source: Implement Sci Commun. 2021 Oct 19;2:120. doi: 10.1186/s43058-021-00222-w (PMC8527650; doi:10.1186/s43058-021-00222-w)
Supplement: Supplementary file 1 — Additional file 1. Initial forward version [file 43058_2021_222_MOESM1_ESM.docx]

**Additional file 1** Initial forward translations

1. CFIR

| **Consolidated Framework for Implementation Research Constructs (CFIR)** | | |
| --- | --- | --- |
| Waltz, Thomas J.; Powell, Byron J.; Fernández, María E.; Abadie, Brenton & Damschroder, Laura J. (2019). Choosing implementation strategies to address contextual barriers: diversity in recommendations and future directions. Implementation science 14(1):42. | | |
| Domäne / Beschreibung | Zugehörige Barriere | |
| **Charakteristika einer Innovation** | | |
| Quelle der Innovation | | Beteiligte haben eine negative Wahrnehmung der Innovation wegen der Instanz, welche die Innovation entwickelt hat und/oder dem Ort, an dem sie entwickelt wurde. |
| Aussagekraft und Qualität der Evidenz | | Beteiligte nehmen die Qualität und die Gültigkeit der zugrundeliegenden Evidenz negativ wahr. |
| Relativer Vorteil | | Beteiligte sehen nicht, inwiefern eine Implementierung der Innovation im Vergleich zu einer alternativen Lösung oder keiner Veränderung einen Vorteil besitzen soll. |
| Anpassbarkeit | | Beteiligte glauben nicht, dass die Innovation adäquat angepasst, verändert oder überarbeitet werden kann, um lokale Bedürfnisse zu erfüllen. |
| Erprobbarkeit | | Beteiligte glauben nicht, dass sie die Innovation in kleinem Umfang innerhalb der Organisation testen können und die Möglichkeit besteht, die Implementierung rückgängig zu machen, falls dies notwendig ist. |
| Komplexität | | Beteiligte glauben, dass die Innovation, wegen Eigenschaften wie Dauer, Wirkungsbereich, Radikalität, Potential Unruhe zu stiften, Veränderung von zentralen Prozessen und Vielschichtigkeit, sowie der Anzahl der benötigten Schritte, komplex ist. |
| Qualität und Präsentation des Designs | | Beteiligte glauben aufgrund der Art, wie die Innovation präsentiert oder gestaltet ist, dass sie von geringer Qualität ist. |
| Kosten | | Beteiligte glauben, dass die Kosten der Innovation und/oder die mit der Implementierung der Innovation verbundenen Kosten (inklusive Investitionen, notwendigem Material und Opportunitätskosten) zu hoch sind. |
| **Umgebung** | | |
| Bedürfnisse und Ressourcen von Patienten | | Bedürfnisse von Patienten, inklusive der Barrieren und Förderfaktoren für die Befriedigung der Bedürfnisse, sind nicht genau bekannt und/oder diese Information ist für die Organisation von geringer Qualität. |
| Weltoffenheit | | Die Organisation ist nicht gut mit anderen Organisationen vernetzt. |
| Druck durch Kollegen | | Es besteht kaum Druck für die Implementierung der Innovation, weil Schlüsselpersonen oder Mitbewerber die Innovation auch noch nicht umgesetzt haben und es aus Gründen des Wettbewerbs nicht notwendig ist. |
| Politische Einflüsse von außen und Anreize | | Politische Einflüsse und Vorschriften von außen (durch die Gesetzgebung oder eine andere zentrale Instanz), externe Mandate, Empfehlungen und Leitlinien, leistungsbezogene Bezahlung, gemeinschaftliche oder öffentliche Berichtspflicht oder Benchmarking existieren nicht, oder untergraben die Anstrengungen zur Implementierung der Innovation. |
| **Handlungsort** | | |
| Strukturelle Charakteristika | | Die soziale Architektur, Alter, Reife und Größe einer Organisation behindert die Implementierung. |
| Netzwerke und Austausch | | Die Organisation besitzt schlechte oder unproduktive soziale Netzwerke und ineffektive formelle und informelle Kommunikationsstrukturen. |
| Kultur | | Kulturelle Normen, Werte und grundlegende Annahmen der Organisation verhindern die Implementierung. |
| Implementierungsklima | | Es besteht kaum Kapazität und schlechte Aufnahmefähigkeit für einen Wandel und es besteht nicht die Annahme, dass die Innovation genutzt, unterstützt oder erwartet wird. |
| Veränderungsdruck | | Beteiligte nehmen die Situation als tolerierbar wahr oder sehen keine Notwendigkeit die Innovation zu implementieren. |
| Kompatibilität | | Die Innovation passt nicht zu den existierenden Arbeitsprozessen und Systemen, zu den existierenden Normen und Werten, zu den Bedürfnissen der Beteiligte oder erhöht das Risiko für sie. |
| Relative Priorität | | Beteiligte glauben, dass andere Initiativen oder Aktivitäten innerhalb der Einrichtung Vorrang haben. |
| Anreize und Prämien der Organisation | | Es existieren kaum konkrete extrinsische Anreize, wie Auszeichnungen für erreichte Ziele, Beförderungen und Gehaltserhöhungen sowie weniger greifbare Anreize wie steigendes Ansehen oder Respekt für die Implementierung der Innovation. |
| Ziele und Feedback | | Ziele werden nicht klar kommuniziert und es wird nicht konsistent nach ihnen gehandelt, Beteiligte erhalten keine Rückmeldungen, die sich auf Ziele beziehen. |
| Lernklima | | Es besteht ein Klima, in dem a) Führungskräfte ihre eigene Fehlbarkeit und Bedürfnisse für Unterstützung und Input durch Teammitglieder nicht ausdrücken; in dem b) Teammitglieder nicht wahrnehmen, dass sie wichtig, wertgeschätzt und kompetente Partner im Veränderungsprozess sind; c) Beteiligte sich nicht sicher fühlen, um neue Methoden auszuprobieren; und d) nicht ausreichend Zeit und Raum für reflexives Denken und Evaluation ist. |
| Bereitschaft für eine Implementierung | | Es gibt kaum konkrete und unmittelbare Hinweise auf eine Bereitschaft oder ein Engagement der Organisation hinsichtlich der Implementierung der Innovation. |
| Engagement der Führungsebene | | Schlüsselpersonen der Führungsebene zeigen kein Engagement, sind nicht involviert oder werden nicht für die Implementierung der Innovation verantwortlich gemacht. |
| Verfügbare Ressourcen | | Ressourcen wie Geld, Räume und Zeit stehen nicht in ausreichendem Umfang zur Verfügung, um die Innovation zu implementieren. |
| Zugang zu Wissen und Informationen | | Beteiligte haben keinen Zugang zu nützlicher Information und Wissen über die Innovation und darüber, wie diese in bestehende Arbeitsaufgaben integriert werden kann. |
| **Charakteristika der Individuen** | | |
| Wissen und Meinungen über die Innovation | | Beteiligte haben negative Einstellungen gegenüber der Innovation, sie schreiben ihr geringen Wert zu und/oder sind mit den Fakten und Prinzipien der Innovation nicht vertraut. |
| Selbstwirksamkeit | | Beteiligte haben kein Vertrauen in ihre eigenen Fähigkeiten, Handlungen so durchzuführen, dass die Implementierungsziele erreicht werden. |
| Individuelles Stadium der Veränderung | | Beteiligte sind nicht qualifiziert oder daran interessiert, die Innovation kontinuierlich zu nutzen. |
| Individuelle Identifikation mit der Organisation | | Beteiligte sind nicht zufrieden mit ihrer Organisation und weisen einen niedrigen Grad an Identifikation mit ihr auf. |
| **Prozesse** | | |
| Planung | | Ein (Ablauf-)Plan mit den notwendigen Schritten zur Implementierung der Innovation wurde nicht oder nur in geringer Qualität entwickelt. |
| Meinungsbildner | | Meinungsbildner (Mitglieder einer Organisation, die formellen oder informellen Einfluss auf die Einstellungen und Überzeugungen ihrer Kollegen in Bezug auf die Implementierung der Innovation haben) sind nicht involviert oder zeigen keine Unterstützung. |
| Offiziell ernannte, intern Verantwortliche für die Implementierung | | Eine qualifizierte Führungskraft (Koordinator, Projektmanager oder Teamleiter) die offiziell die Verantwortung für die Implementierung der Innovation trägt, ist nicht benannt oder zu erkennen. |
| Vorbilder | | Individuen, die als Vorbilder agieren und die Implementierung der Innovation unterstützen, für sie werben oder andere anleiten, so dass Unklarheiten gelöst oder Widerstände bei Schlüsselpersonen überwunden werden, sind nicht involviert oder zeigen keine Unterstützung. |
| Externe Change agents  (externe Beauftragte für Veränderungen) | | Externe Individuen, die formal nominiert sind um Entscheidungen bezüglich der Innovation positiv zu beeinflussen oder zu erleichtern, sind nicht involviert oder zeigen keine Unterstützung |
| Wichtige Interessengruppen | | Vielfältige Strategien zur Gewinnung und Einbeziehung wichtiger Interessengruppen bei der Umsetzung oder Nutzung der Innovation (z. B. durch soziales Marketing, Bildung, Vorbildfunktion, Schulung) sind ineffektiv oder inexistent. |
| Patienten/ Verbraucher | | Vielfältige Strategien zur Gewinnung und Einbeziehung von Patienten/ Verbraucher bei der Implementierung oder Nutzung der Innovation (z. B. durch Social Marketing, Bildung, Vorbildfunktion, Schulung) sind ineffektiv oder inexistent. |
| Ausführung | | Implementierungsaktivitäten werden nicht nach Plan durchgeführt. |
| Reflexion und Evaluation | | Es gibt kaum oder kein quantitatives und qualitatives Feedback über den Fortschritt und die Qualität der Implementierung, es gibt keine regelmäßige Auswertung von Fortschritt und Erfahrung mit Einzelpersonen oder im Team. |

1. ERIC

| **Expertenempfehlungen für die Implementierung einer**  **Veränderung (ERIC)**  Powell, B.J., Waltz, T.J., Chinman, M.J., Damschroder, L.J., Smith, J.L., Matthieu, M.M., Proctor, E.K. & Kirchner, J.E. (2015). A refined compilation of implementation strategies: results from the Expert Recommendations for Implementing Change (ERIC) project. Implementation Science 10:21. | |
| --- | --- |
| Zusammenstellung diskreter ERIC-Implementierungsstrategien (n = 73) | |
| Strategie | Definitionen |
| [1] Neue Finanzierungen abrufen | Neue oder bereits existierende finanzielle Mittel abrufen, um die Implementierung zu erleichtern. |
| [2] Anreiz- / Zulagenstrukturen ändern | Daran arbeiten, eine Anreizstruktur bei der Einführung und Implementierung von klinischen Innovationen zu schaffen. |
| [3] Patienten- / Verbrauchergebühren ändern | Kostenstrukturen schaffen, in denen Patienten / Verbraucher weniger für die bevorzugte Behandlung (die klinische Innovation) bezahlen und mehr für weniger bevorzugte Behandlungen. |
| [4] Bereitschaft beurteilen und Barrieren und Förderfaktoren ermitteln | Verschiedene Aspekte einer Organisation beurteilen, um den Grad der Bereitschaft zur Implementierung zu bestimmen und Barrieren zu identifizieren, die eine Implementierung behindern könnten, sowie Stärken zu identifizieren, die bei den Implementierungsanstrengungen genutzt werden können. |
| [5] Auditieren und Feedback anbieten | Klinische Leistungsdaten über eine spezifische Zeitspanne sammeln und zusammenfassen und dies klinisch Tätigen und der Verwaltung kommunizieren, um das Verhalten von Anbietern zu überwachen, zu evaluieren und anzupassen. |
| [6] Ein Bündnis bilden | Beziehungen mit Partnern für Implementierungsaufgaben aufbauen und pflegen. |
| [7] Lokales Wissen erfassen und teilen | Lokales Wissen in Einrichtungen sammeln, in denen die Implementierung bereits stattgefunden hat, insbesondere dazu, wie klinisch Tätige und jene Personen, die mit der Implementierung beauftragt sind, Veränderungen erfolgreich eingeführt haben und wie sie Ihre Erfahrungen dann mit anderen geteilt haben. |
| [8] Technische Unterstützung zentralisieren | Ein zentrales Systems zur Koordination technischer Unterstützung entwickeln und nutzen, welche für die Implementierung nützlich sind. |
| [9] Akkreditierungs- oder Mitgliedschaftsvoraussetzungen verändern | Sich bemühen, Standards für Zulassungen so zu ändern, dass sie den Einsatz der klinischen Intervention erfordern oder anregen. Voraussetzungen für Mitgliedschaften in der Organisation so verändern, dass zukünftige Mitglieder ermutigt oder genötigt sind, die klinische Innovation zu nutzen. |
| [10] Haftungsrecht ändern | Auf Reformen im Haftungsrecht hinwirken, die unterstützen, dass klinisch Tätige die Innovation bereitwilliger anbieten. |
| [11] Physische Struktur und Ausrüstung ändern | Vorhandene Strukturen evaluieren und, falls notwendig, die physische Struktur und / oder die Ausrüstung so anpassen, dass sie die geplante Innovation bestmöglich unterstützen. |
| [12] Dokumentationssysteme verändern | Dokumentationssysteme so verändern, dass sie eine bessere Beurteilung der Implementierung oder der klinischen Ergebnisse ermöglichen. |
| [13] Angebotsstandort ändern | Den Ort an denen klinischen Dienstleistungen angeboten werden so verändern, dass der Zugang zu ihnen erleichtert wird. |
| [14] Zyklische kleine Tests von Veränderungen durchführen | Veränderungen in sich wiederholender, schrittweiser Art und Weise in begrenztem Umfang einführen, bevor eine systemweite Veränderungen vorgenommen werden. Erhebungen der Veränderungen werden systematisch durchgeführt und Ergebnisse werden hinsichtlich Anzeichen für mögliche Verbesserungen untersucht. Dieser Prozess ist kontinuierlich und wird mit jedem Zyklus verbessert. |
| [15] Bildungsmaßnahmen durchführen | Treffen veranstalten, die auf verschiedene Interessensgruppen (z. B. Anbieter, Führungskräfte, andere Interessensgruppen innerhalb der Organisation, Gesellschaft, Patient/Verbraucher und Angehörige) ausgerichtet sind, um über die klinische Innovation zu informieren. |
| [16] Bildungsmaßnahmen vor Ort durchführen | Eine geschulte Person zur Verfügung stellen, um Anbieter in ihren Praxissettings zu treffen und diese dort in der klinischen Innovation zu schulen, um eine Veränderung zu erreichen. |
| [17] Lokale Konsensusdiskussionen durchführen | Lokale Anbieter und andere Interessensgruppen zu Diskussionen einladen, in denen besprochen wird, inwieweit das gewählte Problem wichtig ist und ob die klinische Innovation angemessen ist, um das Problem zu adressieren. |
| [18] Lokale Bedürfnisse erfassen | Sammeln und analysieren von Daten, die in Bezug zum Innovationsbedarf stehen. |
| [19] Kontinuierliches Training durchführen | Training in der klinischen Innovation in einer nachhaltigen Art und Weise planen und durchführen. |
| [20] Lerngruppen schaffen | Bildung von Lerngruppen unterstützen, in denen sich Anbieter oder anbietende Organisationen zusammenfinden, um sich in einer kollegiale Lernumgebung mit Fragen der Implementierung der klinischen Innovation zu beschäftigen. |
| [21] Neue klinische Teams bilden | Klinische Teams so verändern, dass Personen aus verschiedene Disziplinen und mit unterschiedliche Fähigkeiten zusammenarbeiten, um es wahrscheinlicher zu machen, dass die klinische Innovation angewendet wird (oder erfolgreicher angewendet wird). |
| [22] Qualifizierungsprüfung und/ oder Lizenzstandards schaffen oder verändern | Eine Organisation schaffen, die klinisch Tätige für die Durchführung der Innovation zertifiziert oder eine bestehende Organisation ermutigt, dies zu tun. Die staatliche Berufszulassung oder die entsprechenden Grundlagen so verändern, dass ein Anbieten der Innovation notwendig wird. Darauf hinwirken, dass Fortbildungserfordernisse so verändert werden, dass es wahrscheinlicher wird, dass sich die professionelle Praxis zugunsten der Innovation verändert. |
| [23] Formale Implementierungsvorlage entwickeln | Eine formale Implementierungsvorlage entwickeln, die alle Ziele und notwendigen Strategien beinhaltet. Die Vorlage sollte beinhalten: 1) Ziel/Absicht der Implementierung; 2) Anwendungsbereich der Veränderung (z. B. welche organisatorischen Einheiten betroffen sind); 3) Zeitrahmen und Meilensteine; und 4) dazugehörige Messungen von Leistung und Fortschritt. Den Plan nutzen und aktualisieren, um die Implementierungsbemühungen im Zeitverlauf zu steuern. |
| [24] Akademische Partnerschaften bilden | Eine Partnerschaft mit einer Hochschule oder akademischen Einrichtung anstreben, um gemeinsames Training anzubieten und um Forschungskenntnisse in das Implementierungsprojekt einfließen zu lassen. |
| [25] Implementierungswörterbuch entwickeln | Eine Begriffsliste entwickeln und verteilen, die die Innovation, die Implementierung und die Beteiligten im organisatorischen Wandel beschreibt. |
| [26] Instrumente der Qualitätsüberprüfung entwickeln und implementieren | Qualitätsüberwachungssystemen mit adäquaten Elementen entwickeln, testen und einführen, die spezifisch für die implementierte Innovation sind, wie eine geeignete Sprache, Protokolle, Algorithmen, Standards und Messinstrumente bzgl. Prozesse, Patienten-/ Verbraucherergebnisse und Implementierungsergebnisse. |
| [27] Qualitätsüberwachungssysteme entwickeln und organisieren | Systeme und Prozeduren zur Überwachung klinischer Prozesse und/ oder Ergebnisse zum Zweck der Qualitätssicherung und -verbesserung. |
| [28] Negative Anreize entwickeln | Negative finanzielle Anreize für den Fall des Scheiterns der Implementierung oder für den Fall des Nicht-Nutzens der klinischen Innovationen bereitstellen. |
| [29] Lehrmaterialien entwickeln | Handbüchern und anderen Lehrmaterialen entwickeln, die es den Beteiligten erleichtern, sich über die Innovation zu informieren, und durch welche klinisch Tätige lernen können, wie die klinische Innovation auszuführen ist. |
| [30] Vereinbarungen zur gemeinsamen Nutzung von Ressourcen entwickeln | Partnerschaften mit Organisationen bilden, die über Ressourcen verfügen, die für die Implementierung der Innovation nötig sind. |
| [31] Lehrmaterialien verteilen | Lehrmaterial (einschließlich Richtlinien, Handbücher und Instrumentarien) persönlich, per Post und/ oder elektronisch verteilen. |
| [32] Weitergabe klinischer Daten an die Anbieter fördern | Echtzeitdaten über die wichtigsten Messgrößen für Prozesse und Ergebnisse durch die Verwendung integrierter Kommunikationsmodelle und –kanäle bereitstellen, die die Nutzung der angestrebten Innovation fördern. |
| [33] Förderung | Ein Prozess der interaktiven Problemlösung und Unterstützung, der im Kontext eines erkannten Verbesserungsbedarfs und einer unterstützenden zwischenmenschlichen Beziehung stattfindet. |
| [34] Klinische Innovation finanzieren und Verträge dafür abschließen | Gesetzgeber und andere Kostenträger führen Ausschreibungen durch, um die Innovation anzubieten, nutzen Vertragsgestaltungsprozesse, um Anbieter zur Bereitstellung der klinischen Innovation zu motivieren und entwickeln neue Finanzierungsformen, die er wahrscheinlicher machen, dass Anbieter die Innovation anbieten. |
| [35] Champions identifizieren und bereitstellen | Personen identifizieren und vorbereiten, die sich der Unterstützung, Vermarktung und Durchführung einer Implementierung widmen und Desinteresse oder Widerstände überwinden, die die Innovation in einer Organisation hervorrufen kann. |
| [36] Erstanwender identifizieren | Erstanwender am lokalen Standort identifizieren, um von ihren Erfahrungen mit der Praxisinnovation zu lernen. |
| [37] Nachfrage steigern | Versuchen, den Markt für die klinische Innovation so zu beeinflussen, dass die Wettbewerbsintensität erhöht wird und sich die Bereitschaft des Marktes für die klinische Innovation verbessert. |
| [38] Lokale Meinungsbildner informieren | Leistungsanbieter informieren, die von Kollegen als Meinungsbildner oder einflussreich für die klinische Innovation identifiziert werden, in der Hoffnung, dass sie Kollegen im Wunsch beeinflussen, die Innovation zu übernehmen. |
| [39] Mit Patienten/Verbrauchern intervenieren, um die Inanspruchnahme und die Therapietreue zu fördern | Strategien für Patienten entwickeln, mit dem Ziel, Probleme bezüglich Inanspruchnahme und Therapietreue zu lösen. |
| [40] Führungsausschüsse einbeziehen | Bestehende Führungsstrukturen (z.B. Vorstände, Verwaltungsräte) in die Implementierungsbemühungen einbeziehen, einschließlich führender Personen in die Supervision der Implementierungsprozesse. |
| [41] Patienten/Verbraucher und Familienmitgliedern involvieren | Patienten/Verbraucher und Familien in die Implementierungsbemühungen involvieren. |
| [42] Abrechnung erleichtern | Abrechnungsmodalitäten für die klinische Innovation erleichtern. |
| [43] Training dynamisch machen | Methoden zur Informationsbereitstellung variieren, um auf unterschiedliche Lernstile im Arbeitskontext einzugehen, sowie interaktive Gestaltung des Trainings für die Innovation. |
| [44] Veränderung anordnen | Die Führungsebene die Priorität der Innovation bestätigen lassen und Entschlossenheit ausdrücken lassen, diese zu implementieren. |
| [45] Veränderung modellieren und simulieren | Implementierung der Veränderung vor der eigentlichen Implementierung modellieren und simulieren. |
| [46] Feedback von Patienten/Verbrauchern und der Familie einfordern und nutzen | Strategien entwickeln, um das Feedback der Patienten/ Verbraucher und deren Familien hinsichtlich der Implementierungsbemühungen zu steigern. |
| [47] Formelle Verpflichtungserklärungen einholen | Schriftliche Verpflichtungen von den Schlüsselpartnern einfordern, in denen beschrieben wird, was diese leisten werden, um die Innovation zu implementieren. |
| [48] Treffen des klinischen Implementierungsteams organisieren | Teams bestehend aus klinisch Tätigen bilden und unterstützen, welche die Innovation implementieren; Geschützte Zeit zur Reflexion der Implementierungsbemühungen, des Teilens gewonnener Erkenntnisse und Unterstützung beim gemeinsamen Lernen zur Verfügung stellen. |
| [49] Innovation auf Listen für Einzelleistungsvergütung platzieren | Daran arbeiten, die klinische Innovation in Kataloge aufzunehmen, für welche Anbieter Vergütung erhalten. |
| [50] Patienten/Verbraucher als aktiv Teilnehmende vorbereiten | Patienten/Verbrauchern vorbereiten, sodass diese aktiv an ihrer Versorgung mitwirken, Fragen stellen, und insbesondere sich über Versorgungsleitlinien, die Evidenz hinter klinischen Entscheidungen oder über verfügbare evidenzgestützte Behandlungen erkundigen. |
| [51] Anpassungsfähigkeit fördern | Möglichkeiten der Anpassung einer klinischen Innovation an lokale Bedürfnisse identifizieren und abklären, welche Elemente der Innovation beibehalten werden müssen, um die Wirksamkeit zu gewährleisten. |
| [52] Netzwerkbildung fördern | Bestehende qualitativ hochwertige Arbeitsbeziehungen und Netzwerke innerhalb und außerhalb der Organisation, organisationalen Einheiten, Teams, usw. identifizieren und aufbauen, um den Austausch von Informationen, gemeinsame Problemlösung und Entwicklung gemeinsamer Vorstellungen/Ziele hinsichtlich der Implementierung der Innovation zu fördern. |
| [53] Klinische Supervision bereitstellen | Kontinuierlichen Supervision mit Fokus auf die Innovation für die klinisch Tätigen, einschließlich Training für Supervisoren bereitstellen. |
| [54] Lokale technische Unterstützung anbieten | Ein System für technische Unterstützung entwickeln und nutzen, das sich auf Implementierungsprobleme fokussiert und dabei lokales Personal einsetzt. |
| [55] Kontinuierliche Beratung anbieten | Kontinuierlichen Beratung durch einen oder mehrere Experten für die Implementierungsstrategie und deren Bestandteile anbieten. |
| [56] Implementierung absichtlich erneut prüfen | Fortschritt überwachen und die klinische Praxis und der Implementierungsstrategien anpassen, um die Qualität der Versorgung kontinuierlich zu verbessern. |
| [57] Führungskräfte rekrutieren, benennen und trainieren | Führungskräfte hinsichtlich des Veränderungsprozesses rekrutieren, benennen und schulen. |
| [58] Klinisch Tätige erinnern | Erinnerungssysteme entwickeln, die klinisch Tätige dabei unterstützen, Informationen abzurufen und/oder sie zur Nutzung der klinischen Innovation aufzufordern. |
| [59] Rollen von beruflich Tätigen überarbeiten | Beruflichen Rollen und Tätigkeitsmerkmale verändern oder umgestalten. |
| [60] Andere Experten beobachten („Beschatten“) | Möglichkeiten für Schlüsselpersonen schaffen, erfahrene Personen direkt dabei zu beobachten, wie sie mit gezielten Praxisänderungen/ -innovationen umgehen oder diese anwenden. |
| [61] Bei der Implementierung stufenweise vorgehen | Implementierungsbemühungen phasenweise gestalten, beginnend mit kleinen Pilot- oder Demonstrationsprojekten bis hin zu einer systemweiten Einführung. |
| [62] Eine Organisation zur Dissemination/Verbreitung gründen | Eine separate Organisation oder Einheit identifizieren oder gründen, die für die Verbreitung der klinischen Innovation verantwortlich ist. Es können gewinnorientierte oder gemeinnützige Organisationen sein. |
| [63] Strategien anpassen | Implementierungsstrategien, basierend auf gesammelten Daten anpassen, um Barrieren abzubauen und Förderfaktoren zu unterstützen. |
| [64] Beiräte und Arbeitsgruppen verwenden | Eine formelle Gruppe von mehreren Stakeholdern/Unterstützern bilden und einsetzen, die Input und Beratung zu Implementierungsbemühungen anbieten und Empfehlungen für Verbesserungen geben. |
| [65] Einen Implementierungsberater einsetzen | Unterstützung durch Implementierungsexperten suchen. |
| [66] Pro-Kopf-Vergütung verwenden | Anbieter mit einem festgelegten Betrag pro Patient/Verbraucher für die Bereitstellung der klinischen Versorgung bezahlen. |
| [67] Datenexperten einsetzen | Experten einbeziehen, einstellen und/oder konsultieren, um die Verwendung von Daten während der Implementierungsbemühungen zu optimieren. |
| [68] Data-Warehousing-Techniken verwenden | Klinische Aufzeichnungen über Einrichtungen und Organisationen hinweg integrieren, um die systemübergreifende Implementierung zu erleichtern. |
| [69] Massenmedien verwenden | Medien verwenden, um eine große Anzahl von Menschen zu erreichen, um die Nachricht über die klinische Innovation zu verbreiten. |
| [70] Andere Vergütungsmethoden nutzen | Zahlungsmethoden (in einer Sammelkategorie) einführen. |
| [71] „Train-the-Trainer“ Strategien nutzen | Ausgewählte klinische Tätige oder Organisationen schulen, um andere in der klinischen Innovation zu schulen. |
| [72] Andere Einrichtungen aufsuchen | Einrichtungen aufsuchen, in denen ein ähnlicher Implementierungsansatz erfolgreich war. |
| [73] Mit Bildungseinrichtungen zusammenarbeiten | Bildungseinrichtungen darin bestärken, klinisch Tätige in der Innovation zu schulen. |
