# Supplementary material for: German translation and pre-testing of Consolidated Framework for Implementation Research (CFIR) and Expert Recommendations for Implementing Change (ERIC)
Source: Implement Sci Commun. 2021 Oct 19;2:120. doi: 10.1186/s43058-021-00222-w (PMC8527650; doi:10.1186/s43058-021-00222-w)
Supplement: Supplementary file 2 — Additional file 2. Summary of recommendations by the expert panel [file 43058_2021_222_MOESM2_ESM.docx]

**Additional file 2** Summary of recommendations of expert panel

1. CFIR

1.1 with Helga Breimaier:

| **Item no.*** | **Original item name and no.** | **Recommendations** |
| --- | --- | --- |
| Title | Consolidated Framework for Implementation Research Constructs (CFIR) | Translate “constructs” into German, because it is not included in abbreviation of the instrument. |
| 4 | 1.4 Adaptability | Change “Anpassungsfähigkeit” into “Anpassbarkeit”. |
| Headline | 2. Outer setting | Change „Umgebung“ into „Umfeld“ because „Umgebung“ is more physical but not social. |
| 11 | 2.3 Peer pressure | Change “Druck durch Kollegen” into “Gruppenzwang” because this word is more usual. |
| Headline | 3. Inner setting | Change “Handlungsort” into “Inneres Setting” because “Setting” is usual in German language. |
| 14 | 3.2 Networks and communications | Change “Netzwerk und Austausch” into “Netzwerk und Kommunikation“. |
| 27 | 4.1 Knowledge and beliefs about the intervention | Change “beliefs” into “Überzeugungen” or “Ansichten”. |
| 30 | 4.4 Individual identification with organization | Change “Individuelle Identifikation” in “Identifikation des Einzelnen”. |
| 32 | 5.2 Opinion leaders | Change “Meinungsbilder” into “Meinungsführer”. |
| 33 | 5.3 Formally appointed internal implementation leaders | Change „Offiziell ernannte, intern Verantwortliche für die Implementierung“ into „Formell eingesetzte interne Verantwortliche“. |
| 34 | 5.4 Champions | Change „Vorbilder“ into „Champions“ because “Champions” means more expertise than “Vorbilder”. |
| 35 | 5.5 External Change Agents | Change „Beauftragte für Veränderungen“ into “Berater in der Organisationsentwicklung“ |
| 37 | 5.7 Key Stakeholders | Change „Interessensgruppen“ into „Schlüsselakteure“, because they have an active role. |
| *Assigned unique item number for randomisation during working process. | | |

In General:

- Correct grammar
- Correct punctuation marks

1.2 with Thomas Rotter and Craig Campbell

| **Item no.*** | **Original item name and no.** | **Recommendations** | |
| --- | --- | --- | --- |
| 2 | 1.2 Evidence strength and quality | Change „Aussagekraft“ into „Stärke“; Change „Gültigkeit“ into „Validität“. | |
| Headline | 2. Outer setting | Change „Umgebung“ into „äußere Umgebung“. | |
| Item 9 | 2.1 Patient Needs & Resources | Change „ von geringer Qualität“ into „keine hohe Priorität“. | |
| Headline | Inner setting | Change „Handlungsort“ into „Inneres Setting“, because “Setting” can be used as a German word according to dictionary. | |
| 14 | 3.2 Networks & Communications | Change “Austausch” into “Kommunikation”. | |
| 16 | 3.4 Implementation Climate | Change “Aufnahmefähigkeit” into “Bereitschaft” and „Annahme“ into „Erwartung“. | |
| 17 | 3.5 Tension for Change | Add “gegenwärtige” to translate “present” and keep it closer to the original. | |
| 20 | 3.8 Organizational Incentives & Rewards | Change “kaum konkrete extrinsische” into “keine greifbaren”. | |
| 21 | 3.9 Goals and Feedback | Change „Rückmeldungen“ into „Feedback“ | |
| 24 | 3.12 Leadership Engagement | Add „Manager“. | |
| 26 | 3.14 Access to knowledge and information | Change „nützliche Informationen“ into „leicht verständlich“. | |
| 28 | 4.2 Self-efficacy | Change „Selbstwirksamkeit“ into „Eigene Effizienz“. | |
| 32 | 5.2 Opinion leaders | Change „Meinungsbildner“ into „Meinsungführer“. | |
| 34 | 5.4 Champions | Change „Vorbilder“ into „Champions“. | |
| *Assigned unique item number for randomisation during working process. | | |  |

In General:

- Keep the sentence structure
- Keep the ideas of „and/or“
- Correct punctuation marks
- Correct German grammar

1. ERIC

| **Item no.*** | **Original item name and no.** | **Recommendations** | |
| --- | --- | --- | --- |
| 40 | [1] Access new funding | Change „erleichtern“ into „ermöglichen“. | |
| 41 | [2] Alter incentive/allowance structures | Change „Zuschussstrukturen“ into „Zulagenstrukturen“. | |
| 47 | [8] Centralize technical assistance | Change „Assistenz“ into „Unterstützung“. | |
| 48 | [9] Change accreditation or membership requirements | Change „Zulassung“ into „Akkreditierung“ and „verlangen oder fördern“ into „erfordern oder anregen“. | |
| 49 | [10] Change liability laws | Change „die unterstützen“ into „bereitwilliger“. | |
| 50 | [11] Change physical structure and equipment | Change „physikalisch“ into „baulich“. | |
| 52 | [13] Change service sites | Change „Erreichbarkeit des Angebots verbessern“ into „Angebotsstandort ändern“. | |
| 53 | [14] Conduct cyclical small tests of change | Change „Veränderungen probehalber und in geringem Umfang durchführen“ into „Zyklische Veränderungen in kleinem Umfang durchführen“ and „schreitet ernsthaft fort“ into „kontinuierlich“. | |
| 55 | [16] Conduct educational outreach visits | Change „zu unterstützen“ into „zu schulen“. | |
| 63 | [24] Develop academic partnerships | Change „Zusammenschluss“ into „Partnerschaft“. | |
| 67 | [28] Develop disincentives | Change „Abschreckungsmaßnahmen“ into „Negative Anreize“. | |
| 74 | [35] Identify and prepare champions | Change „Meister“ into „Champions“. | |
| 75 | [36] Identify early adopters | Change „Personen, die… anwenden“ into „Erstanwender“. | |
| 77 | [38] Inform local opinion leaders | Change „Meinungsbilder“ into „Meinungsführer“. | |
| 78 | [39] Intervene with patients/consumers to enhance uptake and adherence | Change „intervenieren“ into „“einschalten“ and „Therapietreue“ into „Adhärenz“. | |
| 83 | [44] Mandate change | Change „Veränderung anordnen“ into „Mandat für Veränderungen“ and „Führungsebene“ into „Leitungsebene“. | |
| 87 | [48] Organize clinician implementation team meetings | Change „Treffen into „Meetings“. | |
| 96 | [57] Recruit, designate, and train for leadership | Change „trainieren“ into „schulen“. | |
| 100 | [61] Stage implementation scale up | Add „stufenweise“. | |
| 103 | [64] Use advisory boards and workgroups | Change „verwenden“ into „nutzen“ and „einsetzen“ into „engagieren“. | |
| *Assigned unique item number for randomisation during working process. | | |  |

In General:

- The English document follows an alphabetical order; generate a logical order in the German document.
- Standardise the sentence structure regarding imperative / infinitive.
