# Supplementary material for: German translation and pre-testing of Consolidated Framework for Implementation Research (CFIR) and Expert Recommendations for Implementing Change (ERIC)
Source: Implement Sci Commun. 2021 Oct 19;2:120. doi: 10.1186/s43058-021-00222-w (PMC8527650; doi:10.1186/s43058-021-00222-w)
Supplement: Supplementary file 3 — Additional file 3. Back-translation [file 43058_2021_222_MOESM3_ESM.docx]

**Additional file 3** Back-translations

1. CFIR

| **Consolidated Framework for Implementation Research Constructs (CFIR)**  Waltz, Thomas J.; Powell, Byron J.; Fernández, María E.; Abadie, Brenton & Damschroder, Laura J. (2019). Choosing implementation strategies to address contextual barriers: diversity in recommendations and future directions. Implementation science 14(1):42. | |
| --- | --- |
| **Domain / Description** | **Associated Barrier** |
| **Characteristics of the intervention** | |
| Source of Intervention | Stakeholders have a negative perception of innovation because of the instance that developed the innovation and/or the place where it was developed. |
| Strength and quality of evidence | Stakeholders have a negative perception of the quality and validity of the underlying evidence. |
| Relative advantage | Stakeholders do not see to what extent an implementation of the innovation should have an advantage compared to an alternative solution or no change. |
| Customizability | Stakeholders do not believe that innovation can be adequately adapted, changed or revised to meet local needs. |
| Testability | Stakeholders do not believe that they can test the innovation on a small scale within the organization and that there is a possibility to reverse the implementation if necessary. |
| Complexity | Stakeholders believe that innovation is complex because of characteristics such as duration, scope, radicality, the potential to create unrest, change of central processes and complexity, and the number of steps required. |
| Quality and presentation of the design | Stakeholders believe that the way the innovation is presented and/or designed means that it is of low quality. |
| Costs | Stakeholders believe that the costs of the innovation and/or the costs associated with the implementation of the innovation (including investments, necessary material and opportunity costs) are too high. |
| **External Setting** | |
| Patient needs and resources | Patient needs, including barriers and facilitators to satisfying needs, are not well known and/or this information is not a high priority for the organization. |
| Open-mindedness | The organisation is not well networked with external organisations. |
| Peer pressure | There is hardly any pressure for the implementation of the innovation because key individuals or competitors have not yet implemented the innovation and it is not necessary for competitive reasons. |
| External policies and incentives | External guidelines, regulations (through legislation or another central body), external mandates, recommendations and guidelines, performance-related pay, collective or public reporting or benchmarking do not exist or undermine efforts to implement innovation. |
| **Internal Setting** | |
| Structural characteristics | The social architecture, age, maturity and size of an organization hinder implementation. |
| Networks and communication | The organisation has poor or unproductive social networks and/or ineffective formal and informal communication structures. |
| Culture | Cultural norms, values and basic assumptions of the organization hinder the implementation. |
| Implementation Climate | There is little capacity and willingness for change and no expectation that innovation will be used, supported or expected. |
| Pressure to change | Stakeholders perceive the current situation as tolerable or see no need to implement the innovation. |
| Compatiability | Innovation does not fit in with existing work processes and systems, with existing norms and values, with the needs of stakeholders or increases the risk for stakeholders. |
| Relative priority | Stakeholders believe that other initiatives or activities within the institution have priority. |
| Incentives and bonuses of the organization | There are no tangible incentives (e.g. awards for objectives achieved, promotions, salary increases) or less tangible incentives (e.g. increasing prestige or respect) for the implementation of innovation. |
| Goals and feedback | Goals are not clearly communicated or acted upon, nor do stakeholders receive feedback related to goals. |
| Learning Climate | There is a climate in which: a) leaders do not express their own fallibility and needs for support and input from team members; b) team members do not perceive that they are important, valued and competent partners in the change process; c) participants do not feel secure in trying out new methods; and d) there is insufficient time and space for reflexive thinking and evaluation. |
| Readiness for implementation | There are hardly any concrete and immediate indications of the willingness or commitment of the organisation to implement the innovation. |
| Management committment | Key executives or executives show no commitment, are not involved or are not held responsible for the implementation of the innovation. |
| Available resources | Resources (e.g. money, space and time) are not sufficiently available to implement the innovation. |
| Access to knowledge and information | Stakeholders do not have adequate access to easily understandable information and knowledge about innovation and how it can be integrated into existing work tasks. |
| **Individual Characteristics** | |
| Knowledge and opinions about the intervention | Stakeholders have negative attitudes towards innovation, attribute little value to it and/or are unfamiliar with the facts and principles of innovation. |
| Self-efficacy | Stakeholders have no confidence in their own ability to perform actions in a way that achieves implementation goals. |
| Individual stage of change | Stakeholders are not qualified or enthusiastic to use the innovation continuously. |
| Individual identification with the organization | Stakeholders are not satisfied with their organization and have a low level of identification with it. |
| **Prozesse** | |
| Planning | A scheme or sequence with the necessary steps to implement the intervention was not developed or only developed in low quality. |
| Opinion leader | Opinion leaders (members of an organisation who have formal or informal influence on the attitudes and convictions of their colleagues regarding the implementation of the intervention) are not involved or show no support. |
| Officially appointed, internally responsible for implementation | A qualified manager (coordinator, project manager or team leader) who is officially responsible for the implementation of the innovation is not named or identified. |
| Champions | Individuals who act as champions and support the implementation of the innovation, promote it or instruct others to resolve ambiguities or overcome resistance from key individuals are not involved or show no support. |
| External Change Agents  (External Change Officer) | External individuals formally nominated to positively influence or facilitate decisions regarding innovation are not involved or do not show support. |
| Important interest groups | A variety of strategies to attract and involve key stakeholders in the implementation or use of innovation (e.g. through social marketing, education, example setting, training) are ineffective or non-existent. |
| Patients/Consumers | A variety of strategies to attract and involve patients/consumers in the implementation or use of innovation (e.g. through social marketing, education, role models, training) are ineffective or non-existent. |
| Execution | Implementation activities are not carried out according to plan. |
| Reflection and Evaluation | There is little or no quantitative and qualitative feedback on the progress and quality of implementation, nor regular evaluation of progress and experience with individuals or in a team. |

1. ERIC

| **Expertenempfehlungen für die Implementierung einer**  **Veränderung (ERIC)**  Powell, B.J., Waltz, T.J., Chinman, M.J., Damschroder, L.J., Smith, J.L., Matthieu, M.M., Proctor, E.K. & Kirchner, J.E. (2015). A refined compilation of implementation strategies: results from the Expert Recommendations for Implementing Change (ERIC) project. Implementation Science 10:21. | |
| --- | --- |
| Zusammenstellung diskreter ERIC-Implementierungsstrategien (n = 73) | |
| Strategy | Definition |
| [1] Call for new financing | Obtain new or existing financial resources to facilitate implementation |
| [2] Change incentive / allowance structures | Work on creating an incentive structure for the introduction and implementation of clinical innovations. |
| [3] Change patient / consumer fees | Create cost structures in which patients/consumers pay less for the preferred treatment (the clinical innovation) and more for less preferred treatments. |
| [4] Assess readiness and identify barriers and support factors | Assess different aspects of an organization to determine the degree of readiness for implementation and identify barriers that may hinder implementation and identify strengths that can be leveraged in the implementation efforts. |
| [5] Audit and provide feedback | Collect and summarize clinical performance data over a specific period of time and communicate this to clinicians and administrators to monitor, evaluate, and adjust provider behavior. |
| [6] Form an alliance | Build and maintain relationships with partners for implementation tasks. |
| [7] Capturing and sharing local knowledge | Gather local knowledge in institutions where the implementation has already taken place, in particular on how clinicians and those responsible for implementation have successfully introduced changes and how they have shared their experiences with others. |
| [8] Centralise technical assistance | Develop and use a central system for coordinating technical support, which is useful for implementation. |
| [9] Change accreditation or membership requirements | Seek to amend standards for approvals so that they require or encourage the use of clinical intervention. Change the requirements for membership in the organization so that future members are encouraged or required to use clinical innovation. |
| [10] Change liability law | Work towards reforms in liability law that encourage clinicians to be more willing to offer innovation. |
| [11] Change physical structure and equipment | Evaluate existing structures and, if necessary, adapt the physical structure and/or equipment to best support the planned innovation. |
| [12] Change documentation systems | Modify documentation systems so that they allow a better assessment of the implementation or clinical results. |
| [13] Change location of the offer | Change the place where clinical services are provided in order to facilitate access to them. |
| [14] Perform cyclical small tests of changes | Introduce changes in a repetitive, step-by-step manner on a limited scale before making system-wide changes. Surveys of the changes are carried out systematically and results are examined for signs of possible improvements. This process is continuous and is improved with each cycle. |
| [15] Carry out educational measures | Organize meetings targeted at different stakeholders (e.g., providers, managers, other stakeholders within the organization, society, patients/consumers and relatives) to inform about clinical innovation. |
| [16] Carry out training measures on site | Provide a trained person to meet providers in their practice settings and train them in clinical innovation to achieve change. |
| [17] Conduct local consensus discussions | Invite local providers and other stakeholders to discuss how important the chosen problem is and whether the clinical innovation is appropriate to address the problem. |
| [18] Identifying local needs | Sammeln und analysieren von Daten, die in Bezug zum Innovationsbedarf stehen. |
| [19] Perform continuous training | Collect and analyse data related to innovation needs |
| [20] Create learning groups | Support the formation of learning groups, in which providers or offering organisations come together to deal with questions concerning the implementation of clinical innovation in a collegial learning environment. |
| [21] Form new clinical teams | Change clinical teams so that people from different disciplines and with different skills work together to make it more likely that the clinical innovation will be applied (or more successfully applied). |
| [22] Create or change qualification tests and/or licensing standards | Establish an organization that certifies clinicians for the implementation of the innovation or encourage an existing organization to do so. Modify the state professional accreditation or the relevant basic principles so that it becomes necessary to offer the innovation. Work to ensure that training requirements are modified to make it more likely that professional practice will change in favour of innovation. |
| [23] Develop formal implementation template | Develop a formal implementation template that includes all goals and necessary strategies. The template should include: 1) goal/objective of implementation; 2) scope of change (e.g. which organizational units are affected); 3) timelines and milestones; and 4) associated measurements of performance and progress. Use and update the plan to manage the implementation efforts over time. |
| [24] Form academic partnerships | Seek a partnership with a university or academic institution to provide joint training and to incorporate research knowledge into the implementation project. |
| [25] Develop an implementation dictionary | Develop and distribute a list of terms that describe innovation, implementation and those involved in organizational change. |
| [26] Develop and implement instruments of quality control | Develop, test and implement quality monitoring systems with adequate elements specific to the innovation implemented, such as appropriate language, protocols, algorithms, standards and measurement tools related to processes, patient/consumer outcomes and implementation results. |
| [27] Develop and organize quality monitoring systems | Systems and procedures for monitoring clinical processes and/or results for the purpose of quality assurance and improvement. |
| [28] Develop negative incentives | Provide negative financial incentives in case of failure of implementation or in case the clinical innovations are not useful. |
| [29] Develop teaching materials | Develop manuals and other educational materials that make it easier for stakeholders to learn about the innovation and for clinicians to learn how to implement the clinical innovation. |
| [30] Develop resource sharing arrangements | Build partnerships with organizations that have the resources needed to implement the innovation. |
| [31] Distribute teaching materials | Distribute teaching materials (including guidelines, manuals and tools) in person, by mail and/or electronically. |
| [32] Encourage the transfer of clinical data to providers | Provide real-time data on key metrics for processes and outcomes through the use of integrated communication models and channels that promote the exploitation of the targeted innovation. |
| [33] Promotion | A process of interactive problem solving and support, which takes place in the context of an identified need for improvement and a supportive interpersonal relationship. |
| [34] Financing clinical innovation and concluding contracts | Legislators and other payers are launching tenders to provide the innovation, using contracting processes to motivate providers to provide the clinical innovation, and developing new forms of financing to make it more likely that providers will provide the innovation. |
| [35] Identify and provide champions | Identify and prepare individuals dedicated to supporting, marketing and executing an implementation, overcoming disinterest or resistance that innovation can create in an organization. |
| [36] Identify first-time users | Identify first-time users at the local site to learn from their experiences with practical innovation. |
| [37] Increase demand | Attempts to influence the demand for clinical innovation in such a way that the intensity of competition is increased and the market maturity of the clinical innovation is improved. |
| [38] Inform local opinion leaders | Inform service providers identified by colleagues as opinion leaders or influential in clinical innovation, in the hope that they will influence colleagues in their desire to adopt the innovation. |
| [39] Switch on patients/consumers to promote utilization and adherence to therapy | Develop strategies with patients to encourage them and solve problems regarding adherence to therapy. |
| [40] Involve management committees | Include existing management structures (e.g. management boards, administrative boards) in the implementation efforts, including the evaluation of the implementation processes. |
| [41] Involve patients/consumers and family members | Involve patients/consumers and families in the implementation efforts. |
| [42] Facilitate accounting | Facilitate billing arrangements for clinical innovation. |
| [43] Making training dynamic | Information delivery methods vary to accommodate different learning styles in the work context, as does the interactive design of training for innovation. |
| [44] Order change | Make the management level understand the priority of innovation and its determination to implement it. |
| [45] Modelling and simulating change | Model or simulate the change to be implemented before the actual implementation. |
| [46] Obtain and use feedback from patients/consumers and their families | Develop strategies to increase feedback from patients/consumers and their families on implementation efforts. |
| [47] Obtain formal commitments | Demand written commitments from key partners describing what they will do to implement the innovation. |
| [48] Organize meetings of the clinical implementation team | Form and support clinical teams that implement the innovation and provide a protected time frame to reflect on the implementation efforts, share experiences and support each other in learning. |
| [49] Put innovation on lists for individual performance remuneration | Work on including clinical innovation in catalogues of single benefit payments for which providers can receive reimbursement (e.g. a drug is included in a formulation, a procedure is now reimbursable) |
| [50] Prepare patients/consumers as active participants | Prepare patients/consumers so that they actively participate in their care, ask questions about care guidelines, inquire about underlying evidence or available evidence-based treatments. |
| [51] Promoting adaptability | Identify ways to adapt a clinical innovation to local needs and clarify which elements of the innovation must be retained to maintain effectiveness. |
| [52] Promote network building | Identify and build existing high quality working relationships and networks within and outside the organisation, organisational units, teams, etc. to promote the exchange of information, joint problem solving and development of common visions/objectives regarding the implementation of the innovation. |
| [53] Provide clinical supervision | Provide continuous supervision to clinicians, with a focus on innovation. Provide training for clinical supervisors who supervise clinicians who offer innovation. |
| [54] Offer local technical support | Develop and use a technical support system that focuses on implementation problems, using local staff. |
| [55] Offer continuous advice | Ongoing advice from one or more experts on the strategies used to support the implementation of the innovation. |
| [56] Deliberately re-check implementation | Monitor progress and adapt clinical practice and implementation strategies to continuously improve the quality of care |
| [57] Recruit, appoint and train managers | Recruiting, appointing and training managers with regard to the change process |
| [58] Remind clinicians | Develop reminder systems designed to help clinicians retrieve information and/or encourage them to use the clinical innovation. |
| [59] Revise roles of professionals | Change or reshape professional roles and job characteristics. |
| [60] Observe other experts ("shadowing") | Create opportunities for key personnel to observe experienced individuals directly as they deal with or apply targeted practice changes/innovations. |
| [61] Proceed step by step with the implementation | Design implementation efforts in phases, starting with small pilot or demonstration projects and moving on to a gradual and system-wide rollout. |
| [62] Founding an organization for dissemination/dissemination | Identify or establish a separate organization or unit responsible for the dissemination of the clinical innovation. These may be for-profit or non-profit organisations. |
| [63] Adapting strategies | Adapt implementation strategies, based on collected data, to reduce barriers and support support factors. |
| [64] Use advisory boards and working groups | Form and engage a formal group of several stakeholders/supporters to provide input and advice on implementation efforts and make recommendations for improvements. |
| [65] Appoint an implementation consultant | Seek support from implementation experts. |
| [66] Use per capita remuneration | compensate providers with a fixed amount per patient/consumer for the provision of clinical care. |
| [67] Use data experts | Involve, hire and/or consult experts to optimize the use of data during implementation efforts. |
| [68] Use data warehousing techniques | Integrate clinical records across facilities and organizations to facilitate cross-system implementation. |
| [69] Use mass media | Use media to reach a large number of people to spread the word about clinical innovation. |
| [70] Use other remuneration methods | Introduce payment methods (in a collection category). |
| [71] Use "Train-the-Trainer" strategies | Train selected clinical practitioners or organizations to train others in clinical innovation. |
| [72] Visit other institutions | visit institutions where a similar implementation approach has been successful. |
| [73] Working with educational institutions | Encourage educational institutions to train clinicians in innovation. |
