# Supplementary material for: German translation and pre-testing of Consolidated Framework for Implementation Research (CFIR) and Expert Recommendations for Implementing Change (ERIC)
Source: Implement Sci Commun. 2021 Oct 19;2:120. doi: 10.1186/s43058-021-00222-w (PMC8527650; doi:10.1186/s43058-021-00222-w)
Supplement: Supplementary file 4 — Additional file 4. Summary of problems found during the pre-testing of the instrument and the modifications proposed [file 43058_2021_222_MOESM4_ESM.docx]

**Additional file 4** Summary of problems found during pre-testing of the instrument and the modifications proposed

1. **CFIR**
   1. **Yellow items**

| **Item no.*** | **Original item name and no.** | **Our German translation** | **Associated heading** | **Problem / proposed modifications** |  |
| --- | --- | --- | --- | --- | --- |
| 2 | 1.2 Evidence Strength & Quality | Stärke und Qualität der Evidenz | Unzureichend wahrgenommene Evidenz / wissenschaftliche Basis | Missing: perception. |  |
| 11 | 2.3 Peer Pressure | Gruppenzwang | Wirtschaftliche Gründe | Missing: intrinsic motivation. |  |
| 15 | 3.3 Culture | Kultur | Organisation | Missing: Specific aspects like values and assumptions. |  |
| 20 | 3.8 Organizational Incentives & Rewards | Anreize und Prämien durch die Organisation | Fehlende Anreize / Gewinn durch die Veränderung | „Prämie“ is confusing because it associated financial. |  |
| 22 | 3.9 Learning Climate | Lernklima | Reflexions-bereitschaft / -fähigkeit | Missing: reflection, communication, esteem. |  |
| 26 | 3.14 Access to knowledge and information | Zugang zu Wissen und Informationen | Einarbeitung | Missing: Somebody showing how to do it. |  |
| 27 | 4.1 Knowledge & Beliefs about the Intervention | Wissen und Überzeugungen über die Innovation | Ablehnung der Innovation / Widerstand gegen die Innovation | Missing: negative attitude. |  |
| 29 | 4.3 Individual Stage of Change | Individuelles Stadium der Veränderung | Voraussetzung der Beteiligten / Charakteristika der Akteure | Missing: the individuals. |  |
| 36 | 5.6 Key Stakeholders | Wichtige Beteiligte | Wirksame Implementierungs-stategien | Missing: Strategies |  |
| 37 (2^nd^) | 5.7 Patients/Customers | Patienten / Verbraucher | Förderung der Partizipation von Nutzern | Missing: aspect of less involvement |  |
| 38 | 5.8 Executing | Ausführung | Nicht-Einhaltung eines Regelwerks | Missing: According to an existing plan. |  |
| *Assigned unique item number for randomisation during working process. | | | | | |

- 1. **Red items**

| **Item no.*** | **Original item name and no.** | **Our German translation** | **Associated heading** | **Problem / proposed modifications** |  |
| --- | --- | --- | --- | --- | --- |
| 10 | 2.2 Cosmopolitanism | Weltoffenheit | Vernetzung | “Weltoffenheit” is something different. |  |
| 10 (2^nd^) | 2.2 Cosmopolitanism | Weltoffenheit | Externe Vernetzung | “Weltoffenheit” is broad, but for this description too open. |  |
| 37 | 5.7 Patients/Customers | Patienten / Verbraucher | Strategien sind nicht effektiv/nicht existent | More central: „Strategien“, not „Patienten/ Verbraucher“ |  |
| *Assigned unique item number for randomisation during working process. | | | | | |

1. **ERIC**
   1. **Yellow items**

| **Item no.*** | **Original item name and no.** | **Our German translation** | **Associated heading** | **Problem / proposed modifications** | |
| --- | --- | --- | --- | --- | --- |
| 40 | [1] Access new funding | Auf neue Finanzierungen zugreifen | Finanzielle / materielle Unterstützung | The word “neu” is inadequate and should be supplemented by “Umwidmung bestehender Mittel”. | |
| 42 | [3] Alter patient/consumer fees | Patienten- / Verbrauchergebühren ändern | Unterschiedliche Kostenstrukturen schaffen |  | |
| 44 | [5] Audit and provide feedback | Auditieren und Feedback anbieten | Datensammlung zur Verhaltens-evaluation | Missing: Providing feedback. | |
| 49 | [10] Change liability laws | Haftungsrecht ändern | Anstoßen von Veränderungen im Haftungsrecht | Confusing if „hinwirken“ or „ändern“, dependent of the audience. | |
| 50 | [11] Change physical structure and equipment | Physische Struktur und Ausrüstung ändern | Evaluation und Anpassung vorhandender Strukturen | Confusing: The word „ändern“. | |
| 51 | [12] Change record systems | Dokumentationssysteme verändern | Optimieren der Dokumentations-systeme | Missing: Optimisation / improvement. | |
| 52 | [13] Change service sites | Angebotsstandort ändern | Zugang zur klinischen Dienstleitungen erleichtern | Confusing if geographic change of a site or inside of a site. | |
| 55 | [16] Conduct educational outreach visits | Bildungsmaßnahmen vor Ort durchführen | Trainer zur klinischen Innovation in der Praxis | MIssing: The clinical innovation. | |
| 57 | [18] Conduct local needs assessment | Lokale Bedürfnisse erfassen | Systematische Problemanalyse | Missing: “Lokal” is only a part of the analysis. “Bedürfnisse” is subjectively. | |
| 58 | [19] Conduct ongoing training | Kontinuierliches Training durchführen | Klinisches Training | More central: Sustainable training, not continuous training. | |
| 60 | [21] Create new clinical teams | Neue klinische Teams bilden | Umstrukturierung hin zu interdisziplinären Teams | Missing: Optimisation of existing teams. Not new teams. | |
| 76 | [37] Increase demand | Nachfrage steigern | Involvierung des Marktes | Missing: The market (macro) is missing. More central: User (micro). | |
| 78 | [39] Intervene with patients / consumers to enhance uptake and adherence | Mit Patienten / Verbrauchern intervenieren, um die Inanspruchnahme und die Therapietreue zu fördern | Problemlösung bei Inanspruch-nahme und Therapietreue | Definition is neutral, but the provided heading has a positive direction (“fördern”) | |
| 79 | [40] Involve executive boards | Führungsausschüsse einbeziehen | Führung in Supervision mit einbeziehen | Missing: Supervision and the active part during implementation. | |
| 82 | [43] Make training dynamic | Training dynamisch machen | Vielfältige Lernzugänge bereitstellen | Missing: Variations of access. | |
| 85 | [46] Obtain and use patients / consumers and family feedback | Feedback von Patienten / Verbrauchern und der Familie einfordern und nutzen | Einbezug von Nutzen | Missing: Use. | |
| 88 | [49] Place innovation on fee for service lists/formularies | Innovation auf Listen für Einzelleistungsvergütung platzieren | Implementierungs-vergütung | The word “Liste” is confusing. | |
| 90 | [51] Promote adaptability | Anpassungsfähigkeit fördern | Anpassung der Intervention | Confusing if adaption or the ability to adapt something. | |
| 93 | [54] Provide local technical assistance | Lokale technische Unterstützung anbieten | Setting spezifisch Unterstützung anbieten | Missing: Implementation. | |
| 96 | [57] Recruit, designate, and train for leadership | Führungskräfte rekrutieren, benennen und trainieren | Ermächtigung der Change Agents | Unclear if head of clinic or the executing health professional is the leader of the innovation. | |
| 99 | [60] Shadow other experts | Andere Experten beobachten („Beschatten“) | Monitoring / Beobachten von Personen | Missing: “Schlüsselpersonen”. | |
| 100 | [61] Stage implementation scale up | Bei der Implementierung stufenweise vorgehen | Implementierungs-phasen | More central: Process in phases, not in steps. | |
| 104 | [65] Use an implementation advisor | Einen Implementierungsberater einsetzen | Unterstützung suchen | Confusing if “Implementierungs-experte” or “-berater” | |
| 106 | [67] Use data experts | Datenexperten einsetzen | Experten nutzen / einbeziehen | The word “Daten” should not be mentioned. | |
| 109 | [70] Use other payment schemes | Andere Vergütungs-methoden nutzen | Aufwand entlohnen | “Andere” is not correct. | |
| 111 | [72] Visit other sites | Andere Einrichtungen aufsuchen | Best practice Beispiele anschauen | Missing: Aspect of successful implementation. | |
| *Assigned unique item number for randomisation during working process. | | | | |  |

- 1. **Red items**

| **Item no.*** | **Original item name and no.** | **Our German translation** | **Associated heading** | **Problem / proposed modifications** |  |
| --- | --- | --- | --- | --- | --- |
| 69 | [30] | Vereinbarungen zur gemeinsamen Nutzung von Ressourcen entwickeln | Implementierungs-förderung durch Netzwerkbildung / Kooperation | Missing: Sharing of ressources. Central: Networking. |  |
| 72 | [33] | Förderung | Interaktiven Behandlungsprozess anregen, unterstützen und fördern | Missing: Too vague. |  |
| *Assigned unique item number for randomisation during working process. | | | | | |
