# Supplementary material for: German translation and pre-testing of Consolidated Framework for Implementation Research (CFIR) and Expert Recommendations for Implementing Change (ERIC)
Source: Implement Sci Commun. 2021 Oct 19;2:120. doi: 10.1186/s43058-021-00222-w (PMC8527650; doi:10.1186/s43058-021-00222-w)
Supplement: Supplementary file 5 — Additional file 5. Final version [file 43058_2021_222_MOESM5_ESM.docx]

**Additional file 5** Final version

1. CFIR

| **Consolidated Framework for Implementation Research Constructs (CFIR)**  Waltz, Thomas J.; Powell, Byron J.; Fernández, María E.; Abadie, Brenton & Damschroder, Laura J. (2019). Choosing implementation strategies to address contextual barriers: diversity in recommendations and future directions. Implementation science 14(1):42. | |
| --- | --- |
| **Domäne / Beschreibung** | **Zugehörige Barriere** |
| **1. Charakteristika der Intervention** | |
| 1.1 Quelle der Intervention | Beteiligte haben eine negative Wahrnehmung der Innovation wegen der Instanz, welche die Innovation entwickelt hat und/oder dem Ort, an dem sie entwickelt wurde. |
| 1.2 Stärke und Qualität der Evidenz | Beteiligte nehmen die Qualität und die Validität der zugrundeliegenden Evidenz negativ wahr. |
| 1.3 Relativer Vorteil | Beteiligte sehen nicht, inwiefern eine Implementierung der Innovation im Vergleich zu einer alternativen Lösung oder keiner Veränderung einen Vorteil besitzen soll. |
| 1.4 Anpassbarkeit | Beteiligte glauben nicht, dass die Innovation adäquat adaptiert, angepasst oder überarbeitet werden kann, um lokale Bedürfnisse zu erfüllen. |
| 1.5 Erprobbarkeit | Beteiligte glauben, dass sie die Innovation in kleinem Umfang innerhalb der Organisation nicht testen können und die Möglichkeit besteht, die Implementierung bei Bedarf rückgängig zu machen. |
| 1.6 Komplexität | Beteiligten glauben, dass die Innovation komplex ist, basierend auf ihrer Wahrnehmung von Dauer, Umfang, Radikalität, Zerstörungskraft, Zentralität und/oder Komplexität und der Anzahl der zur Umsetzung erforderlichen Schritte. |
| 1.7 Qualität und Präsentation des Designs | Beteiligte glauben aufgrund der Art, wie die Innovation gebündelt, präsentiert und/oder gestaltet ist, dass sie von geringer Qualität ist. |
| 1.8 Kosten | Beteiligte glauben, dass die Kosten der Innovation und/oder die mit der Implementierung der Innovation verbundenen Kosten (inklusive Investitionen, Liefer- und Opportunitätskosten) zu hoch sind. |
| **2. Äußeres Setting** | |
| 2.1 Bedürfnisse und Ressourcen von Patienten | Bedürfnisse der Patienten, inklusive der Barrieren und Förderfaktoren für die Befriedigung der Bedürfnisse, sind nicht genau bekannt und/oder diese Information hat für die Organisation keine hohe Priorität. |
| 2.2 Externe Vernetzung (Weltoffenheit) | Die Organisation ist nicht gut mit externen Organisationen vernetzt. |
| 2.3 Gruppenzwang | Es besteht kaum Druck für die Implementierung der Innovation, weil andere Schlüsselpersonen oder konkurrierende Organisationen die Innovation noch nicht umgesetzt haben und die Organisationen dies nicht im Sinne eines Wettbewerbsvorteils tut. |
| 2.4 Externe Richtlinien und Anreize | Externe Richtlinien, Regularien (durch die Gesetzgebung oder eine andere zentrale Instanz), externe Mandate, Empfehlungen und Leitlinien, leistungsbezogene Bezahlung, gemeinschaftliche oder öffentliche Berichtspflicht oder Benchmarking existieren nicht oder untergraben die Anstrengungen zur Implementierung der Innovation. |
| **3. Inneres Setting** | |
| 3.1 Strukturelle Charakteristika | Die soziale Architektur, Alter, Reife und Größe einer Organisation behindern die Implementierung. |
| 3.2 Netzwerke und Kommunikation | Die Organisation besitzt schlechte oder unproduktive soziale Netzwerke und/oder ineffektive formelle und informelle Kommunikationsstrukturen. |
| 3.3 Kultur | Kulturelle Normen, Werte und grundlegende Annahmen der Organisation behindern die Implementierung. |
| 3.4 Implementierungsklima | Es besteht wenig Kapazität für einen Wandel, wenig Bereitschaft und keine Erwartung, dass eine Nutzung der Innovation belohnt, unterstützt oder erwartet wird. |
| 3.5 Veränderungsdruck | Beteiligte nehmen die gegenwärtige Situation als tolerierbar wahr oder sehen keine Notwendigkeit die Innovation zu implementieren. |
| 3.6 Kompatibilität | Die Innovation passt nicht zu den existierenden Arbeitsprozessen und Systemen, zu den existierenden Normen und Werten, zu den Bedürfnissen der Beteiligte oder erhöht das Risiko für Beteiligte. |
| 3.7 Relative Priorität | Beteiligte glauben, dass andere Initiativen oder Aktivitäten innerhalb der Einrichtung Vorrang haben. |
| 3.8 Anreize und Prämien der Organisation | Es existieren keine greifbaren Anreize (z. B. Auszeichnungen für erreichte Ziele, Beförderungen, Gehaltserhöhungen) oder weniger greifbare Anreize (z. B. steigendes Ansehen oder Respekt) für die Implementierung der Innovation. |
| 3.9 Ziele und Feedback | Ziele werden nicht klar kommuniziert oder danach gehandelt, noch erhalten Beteiligte Feedback, das sich auf Ziele bezieht. |
| 3.10 Lernklima | Es besteht ein Klima, in dem a) Führungskräfte ihre eigene Fehlbarkeit und Bedürfnisse für Unterstützung und Input durch Teammitglieder nicht ausdrücken; in dem b) Beteiligte nicht wahrnehmen, dass sie wichtig, wertgeschätzt und kompetente Partner im Implementierungsprozess sind; c) Beteiligte sich psychologisch nicht sicher fühlen, um neue Methoden auszuprobieren; und d) nicht ausreichend Zeit und Raum für reflexives Denken und Evaluation ist. |
| 3.11 Bereitschaft für eine Implementierung | Es gibt wenige greifbare und unmittelbare Hinweise auf eine Bereitschaft oder ein Engagement der Organisation hinsichtlich der Implementierung der Innovation. |
| 3.12 Engagement der Führungsebene | Schlüsselpersonen der Führungsebene oder Führungskräfte zeigen kein Engagement, sind nicht involviert oder werden nicht für die Implementierung der Innovation verantwortlich gemacht. |
| 3.13 Verfügbare Ressourcen | Ressourcen (z. B. Geld, Raum und gewidmete Zeit) stehen nicht in ausreichendem Umfang zur Verfügung, um die Innovation zu implementieren. |
| 3.14 Zugang zu Wissen und Informationen | Beteiligte haben keinen adäquaten Zugang zu leicht verständlicher Information und Wissen über die Innovation und darüber, wie diese in bestehende Arbeitsaufgaben integriert werden kann. |
| **4. Charakteristika der Individuen** | |
| 4.1 Wissen und Überzeugungen über die Intervention | Beteiligte haben negative Einstellungen gegenüber der Innovation, sie schreiben ihr geringen Wert zu und/oder sind mit den Fakten, Wahrheiten und Prinzipien der Innovation nicht vertraut. |
| 4.2 Selbstwirksamkeit | Beteiligte haben kein Vertrauen in ihre eigenen Fähigkeiten, Handlungen so durchzuführen, dass die Implementierungsziele erreicht werden. |
| 4.3 Individuelles Stadium der Veränderung | Beteiligte sind nicht qualifiziert oder enthusiastisch, die Innovation kontinuierlich zu nutzen. |
| 4.4 Individuelle Identifikation mit der Organisation | Beteiligte sind nicht zufrieden mit ihrer Organisation und weisen einen niedrigen Grad an Identifikation mit ihr auf. |
| **5. Prozesse** | |
| 5.1 Planung | Ein Schema oder eine Sequenz mit den notwendigen Schritten zur Implementierung der Intervention wurde nicht oder nur in geringer Qualität entwickelt. |
| 5.2 Meinungsführer | Meinungsführer (Mitglieder einer Organisation, die formellen oder informellen Einfluss auf die Einstellungen und Überzeugungen ihrer Kollegen in Bezug auf die Implementierung der Intervention haben) sind nicht involviert oder zeigen keine Unterstützung. |
| 5.3 Offiziell ernannte, intern Verantwortliche für die Implementierung | Eine qualifizierte Führungskraft (Koordinator, Projektmanager oder Teamleiter), die offiziell die Verantwortung für die Implementierung der Innovation trägt, ist nicht benannt oder zu erkennen. |
| 5.4 Champions | Individuen, die als Champions agieren und die Implementierung der Innovation unterstützen, für sie werben oder andere anleiten, so dass Unklarheiten gelöst oder Widerstände bei Schlüsselpersonen überwunden werden, sind nicht involviert oder zeigen keine Unterstützung. |
| 5.5 Externe Change Agents  (Externe Beauftragte für Veränderungen) | Externe Individuen, die formal nominiert sind, um Entscheidungen bezüglich der Innovation positiv zu beeinflussen oder zu erleichtern, sind nicht involviert oder zeigen keine Unterstützung. |
| 5.6 Wichtige Beteiligte | Vielfältige Strategien zur Gewinnung und Einbeziehung wichtiger Interessengruppen bei der Umsetzung oder Nutzung der Innovation (z. B. durch soziales Marketing, Bildung, Vorbildfunktion, Schulung) sind ineffektiv oder inexistent. |
| 5.7 Patienten/Verbraucher | Vielfältige Strategien zur Gewinnung und Einbeziehung von Patienten/Verbrauchern bei der Implementierung oder Nutzung der Innovation (z. B. durch Social Marketing, Bildung, Vorbildfunktion, Schulung) sind ineffektiv oder inexistent. |
| 5.8 Ausführung | Implementierungsaktivitäten werden nicht nach Plan durchgeführt. |
| 5.9 Reflexion und Evaluation | Es gibt kaum oder kein quantitatives und qualitatives Feedback über den Fortschritt und die Qualität der Implementierung noch eine regelmäßige Auswertung von Fortschritt und Erfahrung mit Einzelpersonen oder im Team. |

1. ERIC

| **Expertenempfehlungen für die Implementierung einer Veränderung (ERIC)**  Powell, B.J., Waltz, T.J., Chinman, M.J., Damschroder, L.J., Smith, J.L., Matthieu, M.M., Proctor, E.K. & Kirchner, J.E. (2015). A refined compilation of implementation strategies: results from the Expert Recommendations for Implementing Change (ERIC) project. Implementation Science 10:21. | |
| --- | --- |
| Zusammenstellung diskreter ERIC-Implementierungsstrategien (n = 73) | |
| **Strategie** | **Definitionen** |
| [1] Auf neue Finanzierungen zugreifen | Auf neue oder bereits existierende finanzielle Mittel zugreifen, um die Implementierung zu erleichtern. |
| [2] Anreiz-/Zulagenstrukturen ändern | Daran arbeiten, eine Anreizstruktur bei der Einführung und Implementierung von klinischen Innovationen zu schaffen. |
| [3] Patienten-/Verbrauchergebühren ändern | Kostenstrukturen schaffen, in denen Patienten/Verbraucher weniger für die bevorzugte Behandlung (die klinische Innovation) bezahlen und mehr für weniger bevorzugte Behandlungen. |
| [4] Bereitschaft beurteilen und Barrieren und Förderfaktoren ermitteln | Verschiedene Aspekte einer Organisation beurteilen, um den Grad der Bereitschaft zur Implementierung zu bestimmen und Barrieren zu identifizieren, die eine Implementierung behindern könnten, sowie Stärken zu identifizieren, die bei den Implementierungsanstrengungen genutzt werden können. |
| [5] Auditieren und Feedback anbieten | Klinische Leistungsdaten über eine spezifische Zeitspanne sammeln und zusammenfassen und diese klinisch Tätigen und der Verwaltung weitergeben, um das Verhalten von Anbietern zu überwachen, zu evaluieren und anzupassen. |
| [6] Ein Bündnis bilden | Beziehungen mit Partnern für Implementierungsaufgaben aufbauen und pflegen. |
| [7] Lokales Wissen erfassen und teilen | Lokales Wissen in Einrichtungen sammeln, in denen die Implementierung bereits stattgefunden hat, insbesondere dazu, wie klinisch Tätige und jene Personen, die mit der Implementierung beauftragt sind, Veränderungen erfolgreich eingeführt haben und wie sie Ihre Erfahrungen dann mit anderen geteilt haben. |
| [8] Technische Unterstützung zentralisieren | Ein zentrales System entwickeln und nutzen, um technische Unterstützung zu liefern, welches für die Implementierung nützlich ist. |
| [9] Akkreditierungs- oder Mitgliedschaftsvoraussetzungen verändern | Sich bemühen, Standards für Zulassungen so zu ändern, dass sie den Einsatz der klinischen Intervention erfordern oder anregen. Voraussetzungen für Mitgliedschaften in der Organisation so verändern, dass zukünftige Mitglieder ermutigt oder genötigt sind, die klinische Innovation zu nutzen. |
| [10] Haftungsrecht ändern | Auf Reformen im Haftungsrecht hinwirken, die unterstützen, dass klinisch Tätige die Innovation bereitwilliger anbieten. |
| [11] Physische Struktur und Ausrüstung ändern | Vorhandene Strukturen evaluieren und, falls notwendig, die physische Struktur und/oder die Ausrüstung so anpassen (z. B. die Gestaltung eines Raumes ändern, Ausrüstung hinzufügen), dass sie die geplante Innovation bestmöglich unterstützen. |
| [12] Dokumentationssysteme verändern | Dokumentationssysteme so verändern, dass sie eine bessere Beurteilung der Implementierung oder der klinischen Ergebnisse ermöglichen. |
| [13] Angebotsstandort ändern | Den Ort an denen klinischen Dienstleistungen angeboten werden so verändern, dass der Zugang zu ihnen erleichtert wird. |
| [14] Zyklische kleine Tests von Veränderungen durchführen | Veränderungen in sich wiederholender, schrittweiser Art und Weise in begrenztem Umfang einführen, bevor systemweite Veränderungen vorgenommen werden. Erhebungen der Veränderungen werden systematisch durchgeführt und Ergebnisse werden hinsichtlich Anzeichen für mögliche Verbesserungen untersucht. Dieser Prozess ist kontinuierlich und wird mit jedem Zyklus verbessert. |
| [15] Bildungsmaßnahmen durchführen | Treffen veranstalten, die auf verschiedene Interessensgruppen (z. B. Anbieter, Führungskräfte, andere Interessensgruppen innerhalb der Organisation, Gesellschaft, Patient/Verbraucher und Angehörige) ausgerichtet sind, um über die klinische Innovation zu informieren. |
| [16] Bildungsmaßnahmen vor Ort durchführen | Eine geschulte Person zur Verfügung stellen, um Anbieter in ihren Praxissettings zu treffen und diese dort in der klinischen Innovation zu schulen, um eine Veränderung zu erreichen. |
| [17] Lokale Konsensusdiskussionen durchführen | Lokale Anbieter und andere Interessensgruppen zu Diskussionen einladen, in denen besprochen wird, inwieweit das gewählte Problem wichtig ist und ob die klinische Innovation angemessen ist, um das Problem zu adressieren. |
| [18] Lokale Bedürfnisse erfassen | Sammeln und analysieren von Daten, die in Bezug zum Innovationsbedarf stehen. |
| [19] Kontinuierliches Training durchführen | Training in der klinischen Innovation in einer nachhaltigen Art und Weise planen und durchführen. |
| [20] Lerngruppen schaffen | Bildung von Lerngruppen unterstützen, in denen sich Anbieter oder anbietende Organisationen zusammenfinden, um sich in einer kollegiale Lernumgebung mit Fragen der Implementierung der klinischen Innovation zu beschäftigen. |
| [21] Neue klinische Teams bilden | Klinische Teams so verändern, dass Personen aus verschiedene Disziplinen und mit unterschiedliche Fähigkeiten zusammenarbeiten, um es wahrscheinlicher zu machen, dass die klinische Innovation angewendet wird (oder erfolgreicher angewendet wird). |
| [22] Qualifizierungsprüfung und/oder Lizenzstandards schaffen oder verändern | Eine Organisation schaffen, die klinisch Tätige für die Durchführung der Innovation zertifiziert oder eine bestehende Organisation ermutigt, dies zu tun. Die staatliche Berufszulassung oder die entsprechenden Grundlagen so verändern, dass ein Anbieten der Innovation notwendig wird. Darauf hinwirken, dass Fortbildungserfordernisse so verändert werden, dass es wahrscheinlicher wird, dass sich die professionelle Praxis zugunsten der Innovation verändert. |
| [23] Formale Implementierungsvorlage entwickeln | Eine formale Implementierungsvorlage entwickeln, die alle Ziele und notwendigen Strategien beinhaltet. Die Vorlage sollte beinhalten: 1) Ziel/Absicht der Implementierung; 2) Anwendungsbereich der Veränderung (z. B. welche organisatorischen Einheiten betroffen sind); 3) Zeitrahmen und Meilensteine; und 4) dazugehörige Messungen von Leistung und Fortschritt. Den Plan nutzen und aktualisieren, um die Implementierungsbemühungen im Zeitverlauf zu steuern. |
| [24] Akademische Partnerschaften bilden | Eine Partnerschaft mit einer Hochschule oder akademischen Einrichtung anstreben, um gemeinsames Training anzubieten und um Forschungskenntnisse in das Implementierungsprojekt einfließen zu lassen. |
| [25] Implementierungswörterbuch entwickeln | Eine Begriffsliste entwickeln und verteilen, die die Innovation, die Implementierung und die Beteiligten im organisatorischen Wandel beschreibt. |
| [26] Instrumente der Qualitätsüberprüfung entwickeln und implementieren | Qualitätsüberwachungssysteme mit adäquaten Elementen entwickeln, testen und einführen, die spezifisch für die implementierte Innovation sind, wie eine geeignete Sprache, Protokolle, Algorithmen, Standards und Messinstrumente bzgl. Prozesse, Patienten-/Verbraucherergebnisse und Implementierungsergebnisse. |
| [27] Qualitätsüberwachungssysteme entwickeln und organisieren | Systeme und Prozeduren zur Überwachung klinischer Prozesse und/oder Ergebnisse zum Zweck der Qualitätssicherung und -verbesserung entwickeln und organisieren. |
| [28] Negative Anreize entwickeln | Negative finanzielle Anreize für den Fall des Scheiterns der Implementierung oder für den Fall des Nicht-Nutzens der klinischen Innovationen bereitstellen. |
| [29] Lehrmaterialien entwickeln | Handbücher und andere Lehrmaterialen entwickeln, die es den Beteiligten erleichtern, sich über die Innovation zu informieren, und durch welche klinisch Tätige lernen können, wie die klinische Innovation auszuführen ist. |
| [30] Implementierung durch Netzwerkbildung/Kooperation fördern (Vereinbarungen zur gemeinsamen Nutzung von Ressourcen entwickeln) | Partnerschaften mit Organisationen bilden, die über Ressourcen verfügen, die für die Implementierung der Innovation nötig sind. |
| [31] Lehrmaterialien verteilen | Lehrmaterial (einschließlich Richtlinien, Handbücher und Instrumentarien) persönlich, per Post und/oder elektronisch verteilen. |
| [32] Weitergabe klinischer Daten an die Anbieter fördern | Echtzeitdaten über die wichtigsten Messgrößen für Prozesse und Ergebnisse durch die Verwendung integrierter Kommunikationsmodelle und –kanäle bereitstellen, die die Nutzung der angestrebten Innovation fördern. |
| [33] Interaktiven Behandlungsprozess anregen, unterstützen und fördern (Förderung) | Ein Prozess der interaktiven Problemlösung und Unterstützung, der im Kontext eines erkannten Verbesserungsbedarfs und einer unterstützenden zwischenmenschlichen Beziehung stattfindet. |
| [34] Klinische Innovation finanzieren und Verträge dafür abschließen | Gesetzgeber und andere Kostenträger führen Ausschreibungen durch, um die Innovation anzubieten, nutzen Vertragsgestaltungsprozesse, um Anbieter zur Bereitstellung der klinischen Innovation zu motivieren und entwickeln neue Finanzierungsformen, die er wahrscheinlicher machen, dass Anbieter die Innovation anbieten. |
| [35] Champions identifizieren und vorbereiten | Personen identifizieren und vorbereiten, die sich der Unterstützung, Vermarktung und Durchführung einer Implementierung widmen und Desinteresse oder Widerstände überwinden, die die Innovation in einer Organisation hervorrufen kann. |
| [36] Erstanwender identifizieren | Erstanwender am lokalen Standort identifizieren, um von ihren Erfahrungen mit der Praxisinnovation zu lernen. |
| [37] Nachfrage steigern | Versuchen, die Nachfrage für die klinische Innovation so zu beeinflussen, dass die Wettbewerbsintensität erhöht wird und sich die Marktreife für die klinische Innovation verbessert. |
| [38] Lokale Meinungsführer informieren | Leistungsanbieter informieren, die von Kollegen als Meinungsführer oder einflussreich für die klinische Innovation identifiziert werden, in der Hoffnung, dass sie Kollegen im Wunsch beeinflussen, die Innovation zu übernehmen. |
| [39] Patienten/Verbraucher involvieren, um die Inanspruchnahme und die Therapietreue zu fördern | Strategien mit Patienten entwickeln, um diese zu ermutigen und Probleme bezüglich Therapietreue zu lösen. |
| [40] Führungsausschüsse einbeziehen | Bestehende Führungsstrukturen (z.B. Vorstände, Verwaltungsräte) in die Implementierungsbemühungen einbeziehen, einschließlich der Bewertung der Implementierungsprozesse. |
| [41] Patienten/Verbraucher und Familienmitglieder involvieren | Patienten/Verbraucher und Familien in die Implementierungsbemühungen involvieren. |
| [42] Abrechnung erleichtern | Abrechnungsmodalitäten für die klinische Innovation erleichtern. |
| [43] Training dynamisch machen | Methoden zur Informationsbereitstellung variieren, um auf unterschiedliche Lernstile im Arbeitskontext einzugehen, sowie interaktive Gestaltung des Trainings für die Innovation. |
| [44] Veränderung anordnen | Die Führungsebene die Priorität der Innovation und ihre Entschlossenheit zu deren Umsetzung erklären lassen, diese zu implementieren. |
| [45] Veränderung modellieren und simulieren | Veränderung modellieren oder simulieren, welche implementiert werden soll, vor der eigentlichen Implementierung. |
| [46] Feedback von Patienten/Verbrauchern und deren Familie einholen und nutzen | Strategien entwickeln, um das Feedback der Patienten/Verbraucher und deren Familien hinsichtlich der Implementierungsbemühungen zu steigern. |
| [47] Formelle Verpflichtungserklärungen einholen | Schriftliche Verpflichtungen von den Schlüsselpartnern einfordern, in denen beschrieben wird, was diese leisten werden, um die Innovation zu implementieren. |
| [48] Treffen des klinischen Implementierungsteams organisieren | Klinische Teams bilden und unterstützen, die die Innovation implementieren und einen geschützten Zeitrahmen geben um die Implementierungsbemühungen zu reflektieren, Erfahrungen zu teilen und sich gegenseitig beim Lernen zu unterstützen. |
| [49] Innovation auf Listen für Einzelleistungsvergütung stellen | Daran arbeiten, die klinische Innovation in Kataloge der Einzelleistungsvergütung aufzunehmen, für welche Anbieter eine Erstattung erhalten können (z. B. wird ein Medikament in eine Formulierung aufgenommen, ein Verfahren ist jetzt erstattungsfähig). |
| [50] Patienten/Verbraucher als aktiv Beteiligte vorbereiten | Patienten/Verbraucher so vorbereiten, dass diese aktiv an ihrer Versorgung mitwirken, Fragen hinsichtlich der Versorgungsleitlinien stellen, die zugrundeliegende Evidenz hinter klinischen Entscheidungen oder über verfügbare evidenzbasierte Behandlungen erkundigen. |
| [51] Anpassungsfähigkeit fördern | Möglichkeiten der Anpassung einer klinischen Innovation an lokale Bedürfnisse identifizieren und Klarheit schaffen, welche Elemente der Innovation beibehalten werden müssen, um die Interventionstreue zu erhalten. |
| [52] Netzwerkbildung fördern | Bestehende qualitativ hochwertige Arbeitsbeziehungen und Netzwerke innerhalb und außerhalb der Organisation, organisationalen Einheiten, Teams, usw. identifizieren und aufbauen, um den Austausch von Informationen, gemeinsame Problemlösung und Entwicklung gemeinsamer Vorstellungen/Ziele hinsichtlich der Implementierung der Innovation zu fördern. |
| [53] Klinische Supervision bereitstellen | Klinisch Tätigen kontinuierliche Supervision zur Verfügung stellen, mit Fokussierung auf die Innovation. Training für klinische Supervisoren anbieten, die Kliniker betreuen, welche die Innovation anbieten. |
| [54] Lokale technische Unterstützung anbieten | Ein System für technische Unterstützung entwickeln und nutzen, das sich auf Implementierungsprobleme konzentriert, unter Nutzung von hiesigem Personal. |
| [55] Kontinuierliche Beratung anbieten | Kontinuierliche Beratung durch einen oder mehrere Experten für die eingesetzten Strategien um die Implementierung der Innovation zu unterstützen. |
| [56] Implementierung absichtlich erneut prüfen | Fortschritt überwachen und die klinische Praxis und der Implementierungsstrategien anpassen, um die Qualität der Versorgung kontinuierlich zu verbessern. |
| [57] Führungskräfte rekrutieren, benennen und schulen. | Führungskräfte hinsichtlich des Veränderungsprozesses rekrutieren, benennen und schulen. |
| [58] Klinisch Tätige erinnern | Erinnerungssysteme entwickeln, die so gestaltet sind, dass sie klinisch Tätige dabei unterstützen, Informationen abzurufen und/oder sie zur Nutzung der klinischen Innovation aufzufordern. |
| [59] Rollen von beruflich Tätigen überarbeiten | Beruflichen Rollen und Tätigkeitsmerkmale verändern oder umgestalten. |
| [60] Andere Experten beobachten („Beschatten“) | Möglichkeiten für Schlüsselpersonen schaffen, erfahrene Personen direkt dabei zu beobachten, wie sie mit gezielten Praxisänderungen/-innovationen umgehen oder diese anwenden. |
| [61] Bei der Implementierung stufenweise vorgehen | Implementierungsbemühungen phasenweise gestalten, beginnend mit kleinen Pilot- oder Demonstrationsprojekten hin zu einer stufenweise und systemweiten Einführung. |
| [62] Eine Organisation zur Dissemination/Verbreitung gründen | Eine separate Organisation oder Einheit identifizieren oder gründen, die für die Verbreitung der klinischen Innovation verantwortlich ist. Es können gewinnorientierte oder gemeinnützige Organisationen sein. |
| [63] Strategien anpassen | Implementierungsstrategien, basierend auf gesammelten Daten anpassen, um Barrieren abzubauen und Förderfaktoren zu unterstützen. |
| [64] Beiräte und Arbeitsgruppen nutzen | Eine formelle Gruppe von mehreren Stakeholdern/Unterstützern bilden und engagieren, die Input und Beratung zu Implementierungsbemühungen anbieten und Empfehlungen für Verbesserungen geben. |
| [65] Einen Implementierungsberater einsetzen | Unterstützung durch Implementierungsexperten suchen. |
| [66] Pro-Kopf-Vergütung verwenden | Anbieter mit einem festgelegten Betrag pro Patient/Verbraucher für die Bereitstellung der klinischen Versorgung vergüten. |
| [67] Datenexperten einsetzen | Experten einbeziehen, einstellen und/oder konsultieren, um das Management über die Verwendung von Daten während der Implementierungsbemühungen informieren. |
| [68] Data-Warehousing-Techniken verwenden | Klinische Aufzeichnungen über Einrichtungen und Organisationen hinweg integrieren, um die systemübergreifende Implementierung zu erleichtern. |
| [69] Massenmedien verwenden | Medien verwenden, um eine große Anzahl von Menschen zu erreichen, um die Nachricht über die klinische Innovation zu verbreiten. |
| [70] Andere Vergütungsmethoden nutzen | Zahlungsmethoden (in einer Sammelkategorie) einführen. |
| [71] „Train-the-Trainer“ Strategien nutzen | Ausgewählte klinische Tätige oder Organisationen schulen, um andere in der klinischen Innovation schulen zu können. |
| [72] Andere Einrichtungen aufsuchen | Einrichtungen aufsuchen, in denen ein ähnlicher Implementierungsansatz erfolgreich war. |
| [73] Mit Bildungseinrichtungen zusammenarbeiten | Bildungseinrichtungen darin bestärken, klinisch Tätige in der Innovation zu schulen. |

69 [30] Implementierung durch Netzwerkbildung / Kooperation fördern (Vereinbarungen zur gemeinsamen Nutzung von Ressourcen entwickeln)

72 [33] Interaktiven Behandlungsprozess anregen, unterstützen und fördern (Förderung)
